# Transcriptomic profiling reveals MEP pathway contributing to ginsenoside biosynthesis in *Panax ginseng*

**DOI:** 10.1186/s12864-019-5718-x

**Published:** 2019-05-17

**Authors:** Le Xue, Zilong He, Xiaochun Bi, Wei Xu, Ting Wei, Shuangxiu Wu, Songnian Hu

**Affiliations:** 1 CAS Key Laboratory of Genome Sciences and Information, Beijing Institute of Genomics, Chinese Academy of Sciences, NO.1 Beichen West Road, Chaoyang District, Beijing, 100101 China; 2 University of Chinese Academy of Sciences, No.19(A) Yuquan Road, Shijingshan District, Beijing, 100049 China; 3 State Key Laboratory of Microbial Resources, Institute of Microbiology, Chinese Academy of Sciences, NO.1 Beichen West Road, Chaoyang District, Beijing, 100101 China

**Keywords:** *Panax ginseng*, RNA-seq, Ginsenoside, MVA pathway, MEP pathway, Terpene precursor

## Abstract

**Background:**

*Panax ginseng* C. A. Mey is one of famous medicinal herb plant species. Its major bioactive compounds are various ginsenosides in roots and rhizomes. It is commonly accepted that ginsenosides are synthesized from terpene precursors, IPP and DMAPP, through the cytoplasmic mevalonate (MVA) pathway. Another plastic 2-C-methyl-D-erythritol 4-phosphate (MEP) pathway was proved also contributing to ginsenoside generation in the roots of *P. ginseng* by using specific chemical inhibitors recently. But their gene expression characteristics are still under reveal in *P. ginseng*. With the development of the high-throughput next generation sequencing (NGS) technologies, we have opportunities to discover more about the complex ginsenoside biosynthesis pathways in *P. ginseng*.

**Results:**

We carried out deep RNA sequencing and comprehensive analyses on the ginseng root samples of 1–5 years old and five different tissues of 5 years old ginseng plants. The de novo assembly totally generated 48,165 unigenes, including 380 genes related to ginsenoside biosynthesis and all the genes encoding the enzymes of the MEP pathway and the MVA pathway. We further illustrated the gene expression profiles related to ginsenoside biosynthesis among 1–5 year-old roots and different tissues of 5 year-old ginseng plants*.* Particularly for the first time, we revealed that the gene transcript abundances of the MEP pathway were similar to those of the MVA pathway in ginseng roots but higher in ginseng leaves. The IspD was predicated to be the rate-limiting enzyme in the MEP pathway through both co-expression network and gene expression profile analyses.

**Conclusions:**

At the transcriptional level, the MEP pathway has similar contribution to ginsenoside biosynthesis in ginseng roots, but much higher in ginseng leaves, compared with the MVA pathway. The IspD might be the key enzyme for ginsenoside generation through the MEP pathway. These results provide new information for further synthetic biology study on ginsenoside metabolic regulation.

**Electronic supplementary material:**

The online version of this article (10.1186/s12864-019-5718-x) contains supplementary material, which is available to authorized users.

## Background

Asian ginseng, *Panax ginseng* C. A. Mey belonging to the genus *Panax* and the family Araliaceae, is one of famous medicinal and deciduous perennial plant species and widely distributes in China, Japan and Korea [1]. Its medicinal function was first reported in the Chinese medicine monograph, *Shennong Herbal Classics*, in about 3500 BC, which has over 5000-year history [2]. According to the modern pharmacological research, *P. ginseng* has various biological activities, such as antistress, antihypertensive, antivirus, antitumor, and immune modulatory activities, so it is not only widely used to cure central nervous system diseases and chronic diseases [1, 3], but also used to fight against cancer and resist aging [2, 4]. Therefore, it is known as the king of herbs and its genus name, *Panax*, means “cure all” in Greek [4]. The present economic value of *P. ginseng* in the global medicinal plant trade is estimated to be in excess of $2.1 billion [5].

The main medicinal parts of *P. ginseng* are its roots and rhizomes. The major bioactive components are ginsenosides, kinds of triterpene saponins, including oleanane-type ones and dammarane-type ones, based on their chemical skeleton structures [6]. The precursors of these active compounds are terpenoids, which are the largest class of secondary metabolites produced by plants. Based on previous studies, ginsenoside biosynthesis process consists of three stages: terpene precursor biosynthesis, triterpene backbone biosynthesis and various ginsenoside generation. The first stage is to generate two types of terpene precursors, isopentenyl pyrophosphate (IPP) and its isomer, dimethylallyl pyrophosphate (DMAPP) [7], through two isoprenoid biosynthetic pathways, mevalonate (MVA) pathway and 2-C-methyl-D-erythritol 4-phosphate (MEP) pathway, respectively, depending on the organism species [8, 9]. Because plants commonly have both cytosolic MVA pathway and chloroplastic MEP pathway for IPP and DMAPP synthesis, while green algae and cyanobacteria predominantly contain the MEP pathway [10–12]. So far, it is accepted that the MVA pathway participates in the ginsenoside biosynthesis in *Panax* genus based on both phytochemical experiments and transcriptome and genome sequencing studies [13–18]. Until 2014, Zhao and coworkers reported the MEP pathway also involved in ginsenoside biosynthesis by using inhibitor experiments on ginseng hairy roots [19]. But there is no detail report about the genes and gene expression characteristics of the MEP pathway in *P. ginseng* except that recent Chen’s group found there were genes of the MEP pathway annotated in the genome sequencing study of *P. ginseng* [20]. In *Arabidopsis thaliana* [21] and *Hevea brasiliensis* (rubber tree) [22, 23], it was reported that the genes of the MVA pathway and MEP pathway both involving in the biosynthesis of IPP and DMAPP based on their genome sequencing studies. Therefore, with the development of the high-throughput next generation sequencing (NGS) technologies, we have opportunities to discover more genomic information about the complex biosynthesis pathways of ginsenoside and their related gene expression characteristics in *P. ginseng*.

In cultivation condition, 5 years old ginseng plants are usually regarded as mature enough to harvest for medicinal utilization. In this study, we carried out deep RNA sequencing both on root samples of 1–5 years old and other tissues (including leaves, roots, stems, rhizomes, root cores) of 5 years old *P. ginseng* (Additional file 6: Figure S1) collected in field in the ginseng farm in northeast China, and further focused the bioinformatic analyses on gene annotation and expression profiles related to ginsenoside biosynthesis among these tissues. We totally identified 380 genes encoding enzymes involved in triterpene saponin biosynthesis. Furthermore, we for the first time annotated all the genes involved in the MEP pathway in *P. ginseng*, as well as revealed their transcriptions had almost equal abundance to those of the MVA pathway in the terpene precursor biosynthesis process in the roots of *P. ginseng*. But in the leaves of 5-year old *P. ginseng*, the gene expression levels of the MEP pathway were highest compared with those in other tissues of *P. ginseng*. We also predicted the key enzyme in the MEP pathway was 2-C-methyl-D-erythritol 4-phosphate cytidylyl transferase (IspD) using co-expression network analysis. Our results provide a new insight and bioinformatic basis for further synthetic biology study on metabolic regulation of ginsenoside biosynthesis in the future.

## Results

### Quality control and de novo assembly of RNA-seq reads

In this work, the sequencing on each ginseng mRNA sample generated more than 20 million reads (Additional file 1: Table S1). After filtering adaptors, low quality reads, rRNA reads, mitochondrial and plastic RNA reads and bacterial reads, we got high-quality clean reads, accounting for more than 85% of the raw reads.

The sequencing saturation of each RNA-seq sample (Additional file 7: Figure S2) and the distribution of gene expression levels (Additional file 8: Figure S3) were also assayed in order to make sure the RNA-seq data meeting the quality for further analyses.

The correlation analyses between two biological replicates of the RNA-seq samples among 1–5 years old ginseng root tissues were conducted and the results showed that the values of correlation coefficient R, particularly based on the genes of the MEP pathway, were all above 0.85 (Additional file 9: Figure S4). Though these R values were not as high as those of indoor experimental results due to the environmental influence on sampling in field conditions in this study, further heatmap clustering analyses on gene expressions among all the root RNA-seq samples showed that two root replicates of each age clustered together (Additional file 10: Figure S5), indicating that the correlation between the two biological replicates is acceptable.

After assembling the clean high quality reads based on all the RNA-seq samples merged together, the data totally generated 48,165 unigenes, with the contig N50 size of 1667 bp and average contig size of 1268 bp. The longest isoforms’ contig N50 size is 1714 bp and an average size is 1242 bp (Table 1), which is shorter than that of the total assembled genes because the later one was statisticated based on the gene expression value (TPM > =1).

**Table 1:** Summary of quality statistics of Trinity assembly of the RNA-seq data of *P. ginseng*

Trinity assembly	Sequence
Counts of transcripts	
Total trinity genes	48,165
Total trinity transcripts	84,542
Percent GC (%)	40.73
Stats based on all transcript contigs	
Contig N50 (bp)	1667
Median contig length (bp)	1060
Average contig (bp)	1268.41
Total assembled bases (bp)	107,234,113
Stats based on only longest isoform per gene	
Contig N50 (bp)	1714
Median contig length (bp)	992
Average contig (bp)	1242.54
Total assembled bases (bp)	59,846,708

(Note: The gene number was statisticated based on the gene expression abundance of TPM > =1)

### Identification of candidate genes encoding enzymes involved in ginsenoside biosynthesis

According to previous studies, the ginsenoside biosynthesis process includes three metabolic modules: terpene precursor synthesis, triterpene skeleton synthesis and various-type ginsenoside synthesis [13, 24, 25]. The terpene precursor biosynthesis module consists the MVA pathway and the MEP pathway to generate terpene precursor, IPP or DAMPP [8, 9]. Based on our annotation results, the genes encoding the enzymes involved in the three metabolic modules were all retrieved from our assembled ginseng RNA-seq datasets (Table 2). Particularly for the MEP pathway, it was for the first time to identify all the genes along the whole pathway based on transcriptomic data of ginseng samples. Its largest enzyme family was 1-deoxy-D-xylulose-5-phosphate synthase (DXS) and consisted of about 13 sub-families, 20 genes and 56 isoforms, while its smallest enzyme family was 2-C-methyl-D-erythritol 4-phosphate cytidylyl transferase (IspD), only 2 sub-families, 2 genes and 10 isoforms identified. In addition, 3-hydroxy-3-methylglutaryl-CoA reductase (HMGR) in the MVA pathway, geranylgeranyl diphosphate synthase (GGR) in the triterpene skeleton biosynthesis module, as well as glycosyl transferase (GT) and cytochrome P450 (CYP450) in the ginsenoside biosynthesis module were also found being the largest protein family in their corresponding metabolic modules, respectively. Furthermore, the gene diversity happened not only in root tissues but also in other tissues of ginseng plants (Table 2).

**Table 2:** Statistics on the gene numbers related to ginsenoside biosynthesis in the RNA-seq samples of *P. ginseng*

		In ginseng roots of 1–5 years old				In different ginseng tissues of 5 years old			
Pathway	Enzyme	Sub-family	Cluster	Gene	Isoform	Sub-family	Cluster	Gene	Isoform
MVA	AACT	6	8	8	8	9	11	11	18
	HMGS	8	8	9	16	11	12	12	15
	HMGR	13	14	17	27	16	17	19	28
	MVK	2	2	2	12	2	2	3	13
	PMK	8	8	19	26	5	5	16	19
	MVD	2	2	2	2	2	2	2	2
MEP	DXS	13	14	20	56	10	10	11	37
	DXR	8	8	8	13	7	7	7	11
	IspD	2	2	2	7	2	2	2	6
	IspE	4	4	4	6	2	2	2	2
	IspF	2	2	4	10	2	2	4	10
	IspG	3	3	3	14	2	2	2	10
	IspH	7	7	7	10	7	7	7	10
Skeleton synthesis	IPI	2	2	2	3	5	5	5	6
	GGR	12	14	15	31	12	13	15	26
	FPS	2	2	2	14	3	3	3	14
	SS	1	2	2	13	1	2	2	10
Saponin synthesis	SE	8	10	10	21	9	11	11	20
	DS	2	3	3	6	3	4	4	6
	AS	7	7	8	13	4	4	4	12
	GT	105	112	135	226	103	110	133	231
	CYP450	81	93	98	188	80	89	93	181

### The dynamic gene expression profiles related to ginsenoside biosynthesis in *P. ginseng*

**Fig. 1:**
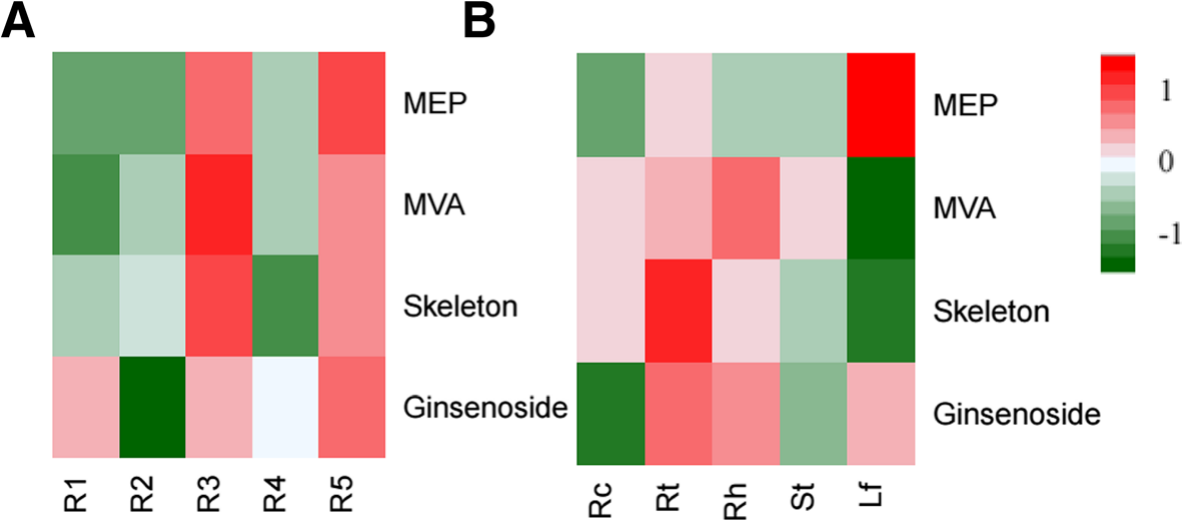
Gene expression profiles related to three metabolic pathway modules of ginsenoside biosynthesis of *P. ginseng*. **a**, the profiles in ginseng roots of 1–5 years old. **b**, the profiles in different ginseng tissues of 5 years old. MEP, the MEP pathway. MVA, the MVA pathway. Skeleton, the triterpene skeleton biosynthesis pathway. Ginsenoside, the late steps for ginsenoside biosynthesis pathway. R1-R5, ginseng root samples of 1–5 years old. Rc, root cores. Rt, lateral roots. Rh, rhizomes. St, stems, Lf, leaves. For the calculation of the gene expression levels of each pathway module of each sample, all the TPM values of the expressed gene annotated to the enzymes of this module of that sample were summarized together. The heatmap was drawn with R package geneplotter and gplots

According to the heatmap of gene expression profiles related to ginsenoside biosynthesis (Fig. 1), we could see that the gene transcript abundances in all the three metabolic modules were obviously higher in the ginseng roots of 3 years old and 5 years old, but not in the roots of 4 years old (Fig. 1a). This inconstancy might be due to sampling bias in field conditions and would be validated by experiments in the future. On the other hand, for the different tissues of 5 years old ginseng, the genes of the MEP pathway had the highest expression level in the leaves, followed by in the roots, rhizomes, stems and lowest in the root cores (Fig. 1b). On the contrary, the genes of the MVA pathway had the highest expression levels in ginseng rhizomes, followed by in the roots, root cores, stems, and lowest in the leaves. For the triterpene skeleton synthesis pathway, its gene transcription levels were highest in ginseng roots, relative higher in the rhizomes, root cores and stems, but lowest in the leaves. For the genes of ginsenoside synthesis pathway module, their expression levels were highest in the roots, followed by in the rhizomes, leaves and lowest in the root cores (Fig. 1b).

Therefore, the gene transcription levels related to the MVA pathway, triterpene skeleton biosynthesis and the final steps of ginsenoside biosynthesis were generally higher in the underground tissues, especially in the roots and rhizomes of *P. ginseng*. But the genes involved in the MEP pathway transcribed at high levels predominantly in the leaves, followed by in the lateral roots, but at low levels in the vascular and xylem tissues (root cores and stems), whose phenomenon consisted with its plastic origin [9]. The gene expression levels in the final steps of ginsenoside biosynthesis, mostly consisting of GT and CYP450 super gene families, were also higher in the living cells (such as in the lateral roots, rhizomes and leaves), but much lower in the vascular and xylem cells (such as in the root cores and stems) of ginseng plants (Fig. 1b).

### Gene expression profiles reveal the MEP pathway contributing to saponin synthesis in ginseng

**Fig. 2:**
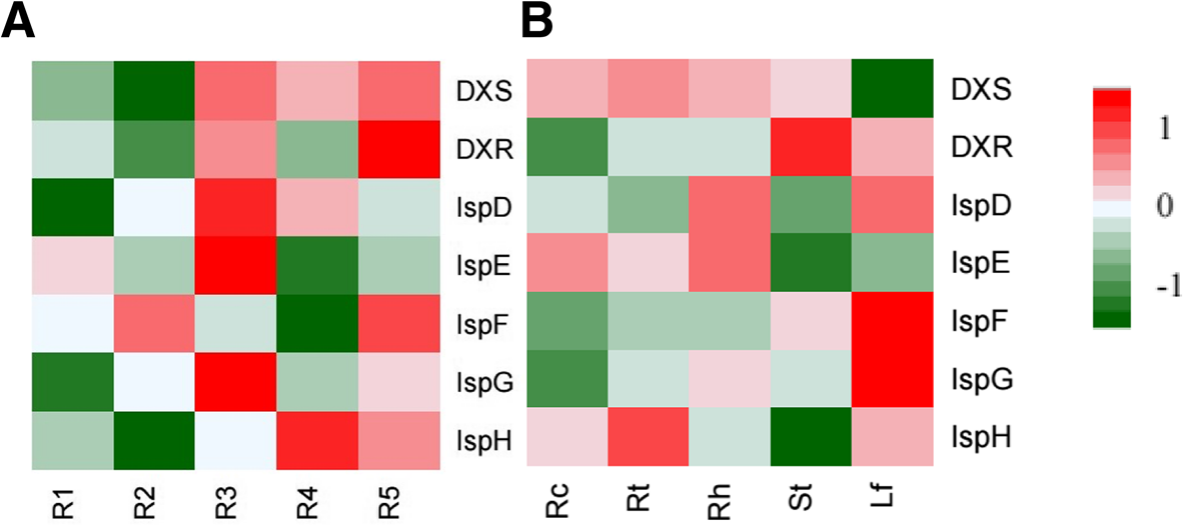
Gene expression profiles related to the MEP pathway of *P. ginseng*. (**a**), the profiles in ginseng roots of 1–5 years old. **b**, the profiles in different ginseng tissues of 5 years old. R1-R5, ginseng root samples of 1–5 years old. Rc, root cores. Rt, lateral roots. Rh, rhizomes. St, stems, Lf, leaves. DXS, DXR, IspD, IspE, IspF, IspG and IspH, the genes in the MEP pathway. AACT, HMGS, HMGR, MVK, PMK and MVD, the genes in the MVA pathway. For the calculation of each gene expression levels in the MEP pathway of each sample, all the TPM values of the expressed isoforms annotated to that gene of that sample were summarized together. The heatmap was drawn with R package geneplotter and gplots

The gene expression characteristics of the MEP pathway of *P. ginseng* were further inspected among different tissues of 5 years old ginseng plants and 1–5 year-old ginseng root samples. Actually, the transcript abundances of each gene’s isoforms were variant and changed in different tissues during ginseng growing up (Additional file 11: Figure S6). Therefore, we used the total transcript abundances of all the isoforms of each gene to be that gene’s transcription level. The results showed the MEP-pathway genes transcribed divergently in different tissues of *P. ginseng* (Fig. 2). In the roots, the genes expressed at much higher levels in 3–5 years old ginseng plants than those of 1–2 years old ones, especially for the genes DXS, DXR and IspH (Fig. 2a). But for different tissues of 5 years old ginseng plants, more genes transcribed at higher levels in ginseng leaves than in other tissues, except for two enzymes, DXS and IspE (Fig. 2b).

**Fig. 3:**
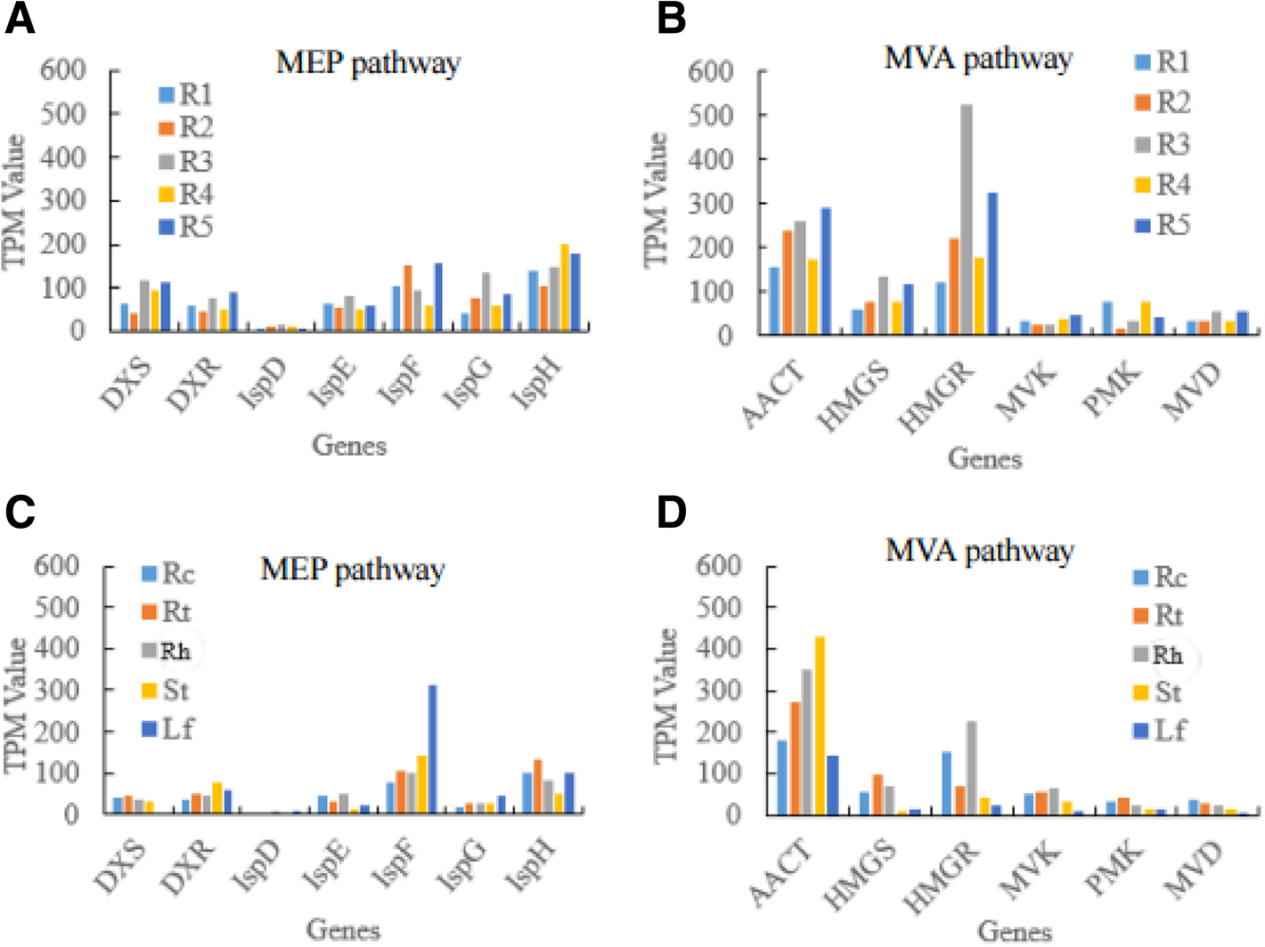
The expression levels (TPM) of the genes involved in the MEP pathway and the MVA pathway of *P. ginseng* based on RNA-seq analyses. **a**, genes in the MEP pathway of the ginseng root samples of 1–5 years old. **b**, genes in the MVA pathway of the ginseng root samples of 1–5 years old. **c**, genes in the MEP pathway of different tissues of 5 year-old ginseng. **d**, genes in the MVA pathway of different tissues of 5 year-old ginseng. R1-R5, ginseng root samples of 1–5 years old. Rc, root cores. Rt, lateral roots. Rh, rhizomes. St, stems, Lf, leaves. DXS, DXR, IspD, IspE, IspF, IspG and IspH, the genes in the MEP pathway. AACT, HMGS, HMGR, MVK, PMK and MVD, the genes in the MVA pathway. For the calculation of each gene transcription levels of each sample, all the TPM values of the expressed isoforms annotated to that gene of that sample were summarized together

Further detection and comparison on the gene transcript abundance between the MEP pathway and the MVA pathway (Fig. 3a, b) showed that the gene transcription levels of the two pathways in general were not significantly different in ginseng root samples, indicating that the MEP pathway also contributed to ginsenoside synthesis in the roots of *P. ginseng*. However, the IspD gene of the MEP pathway transcribed at very low abundance no matter in ginseng roots or in other ginseng tissues (Fig. 3a, c), while the MVK, PMK and MVD genes of the MVA pathway also transcribed at low levels in all the ginseng tissue samples (Fig. 3b, d). These four low-transcribed genes might have essential impact on IPP or IPP-derived metabolite generation, speculating that they might be the potential targets for metabolic manipulation of ginsenoside production using synthetic biology techniques in the future.

### Gene expression profiling of the whole ginsenoside biosynthesis route in *P. ginseng*

**Fig. 4:**
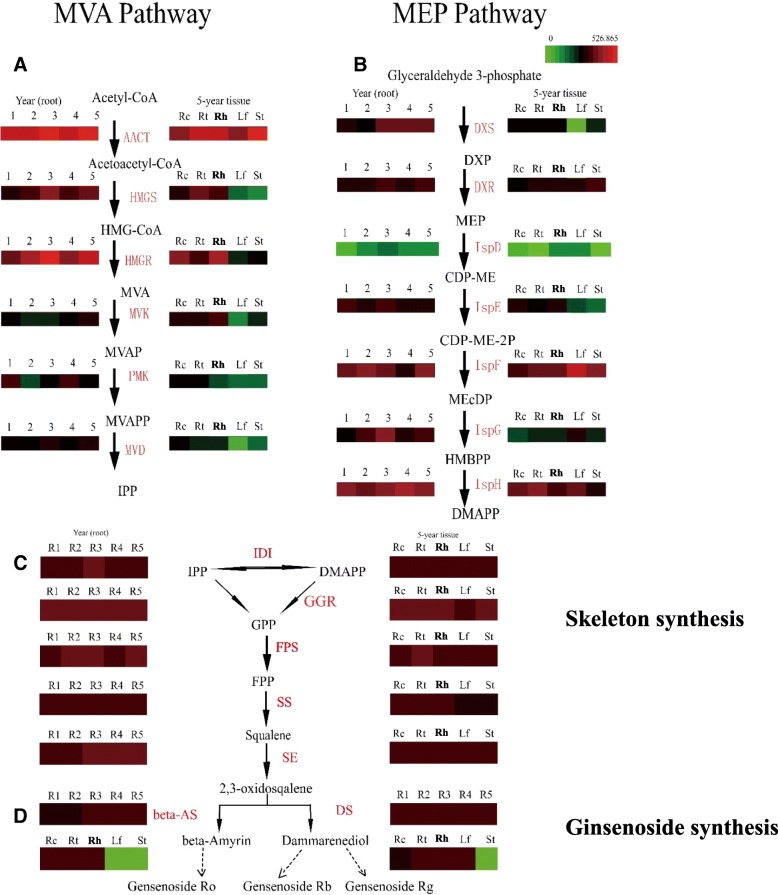
The whole gene expression patterns related to ginsenoside biosynthesis in *P. ginseng*. **a**, the MVA pathway. **b**, the MEP pathway. **c**, the triterpene skeleton biosynthesis pathway. **d**, the late steps of ginsenoside biosynthesis pathway. DXS, DXR, IspD, IspE, IspF, IspG and IspH, the genes in the MEP pathway. AACT, HMGS, HMGR, MVK, PMK and MVD, the genes in the MVA pathway. IDI, isopentenyl pyrophosphate isomerase. GGR, FPS, SS and SE, the genes in the pathway of triterpene skeleton biosynthesis. Β-AS, β-amyrin synthase. DS, dammarenediol-II synthase. GT, glycosyltransferase. CYP450, cytochrome P450.R1-R5, ginseng root samples of 1–5 years old. Rc, root cores. Rt, lateral roots. Rh, rhizomes St, stems, Lf, leaves. For the calculation of each gene expression levels in each pathway of each sample, all the TPM values of the expressed isoforms annotated to that gene of that sample were summarized together. The heatmap was drawn with R package geneplotter and gplots

To show the comprehensive gene expression characteristics of the whole ginsenoside biosynthesis route in *P. ginseng*, we draw the gene expression profiles based on TPM values of the transcribed genes from a whole point of view of all the three metabolic modules in both ginseng roots of 1–5 years old and different ginseng tissues of 5 years old, respectively. The total gene expression patterns along the whole pathway were drawn in Fig. 4.

From the whole point of view, we could see that the transcription of IspD gene of the MEP pathway was obviously at a very low level, not only in ginseng roots of 1–5 years old but also in the different mature ginseng tissues of 5 years old, indicating the IspD might be the limitation enzyme in the MEP pathway for saponin biosynthesis in *P. ginseng*. Moreover, the genes in the first 4 steps of the MEP pathway transcribed at relatively lower levels compared with those in the late 3 steps (Fig. 4b). On the contrary, the genes involved in the first 3 steps of the MVA pathway expressed at higher levels compared with those in the late 3 steps (Fig. 4a). In addition, except for the first gene AACT, other genes of the MVA pathway transcribed at lower levels in the stems and leaves compared with other tissues of 5-year-old ginseng plants, consistent with above results that the MVA pathway mainly expressed in the underground tissues of *P. ginseng* (Figs. 1 and 3b, d).

The terpene precursors, IPP and DMAPP, either generated by the MVA pathway or the MEP pathway, can be transformed each other by IDI then go into the triterpene skeleton biosynthesis pathway. Based on the gene expression profiling from the whole point of view, the genes of the triterpene skeleton synthesis pathway transcribed constantly among all the tissues and all the ages in our ginseng samples (Fig. 4), showing that they might have little suppression effect on ginsenoside biosynthesis.

For various ginsenoside biosynthesis, the triterpene skeleton, 2,3-oxidosqualene is catalyzed by dammarenediol synthase (DS) to form dammarenediol II and finally to generate dammarane-type ginsenosides (including ginsenoside Rb and Rg) [26],or is catalyzed by β-amyrin synthase (β-AS) to form β-amyrin to terminally produce oleanene-type ginsenosides (ginsenoside Ro) [6]. These triterpenes are converted to various ginsenosides via hydroxylation by cytochrome P450 (CYP450) enzymes or putatively via subsequent glycosylation by glycosyltransferases (GT) in the terminal steps of ginsenoside synthesis pathway [26]. From our results, the gene encoding β-AS transcribed at very low levels in the leaves of *P. ginseng*, while the gene encoding DS expressed at relative high levels both in the roots, rhizomes and leaves of *P. ginseng*, indicating ginsenoside Ro’s synthesis probably mainly occurred in the tissues of underground but ginsenoside Rb and Rg could be synthesized in the whole ginseng plant. This discovery also gives inspiration on metabolic regulation of ginsenoside production using synthetic biological methods in the future, but needs further experimental confirmation.

### The key gene of MEP pathway

**Fig. 5:**
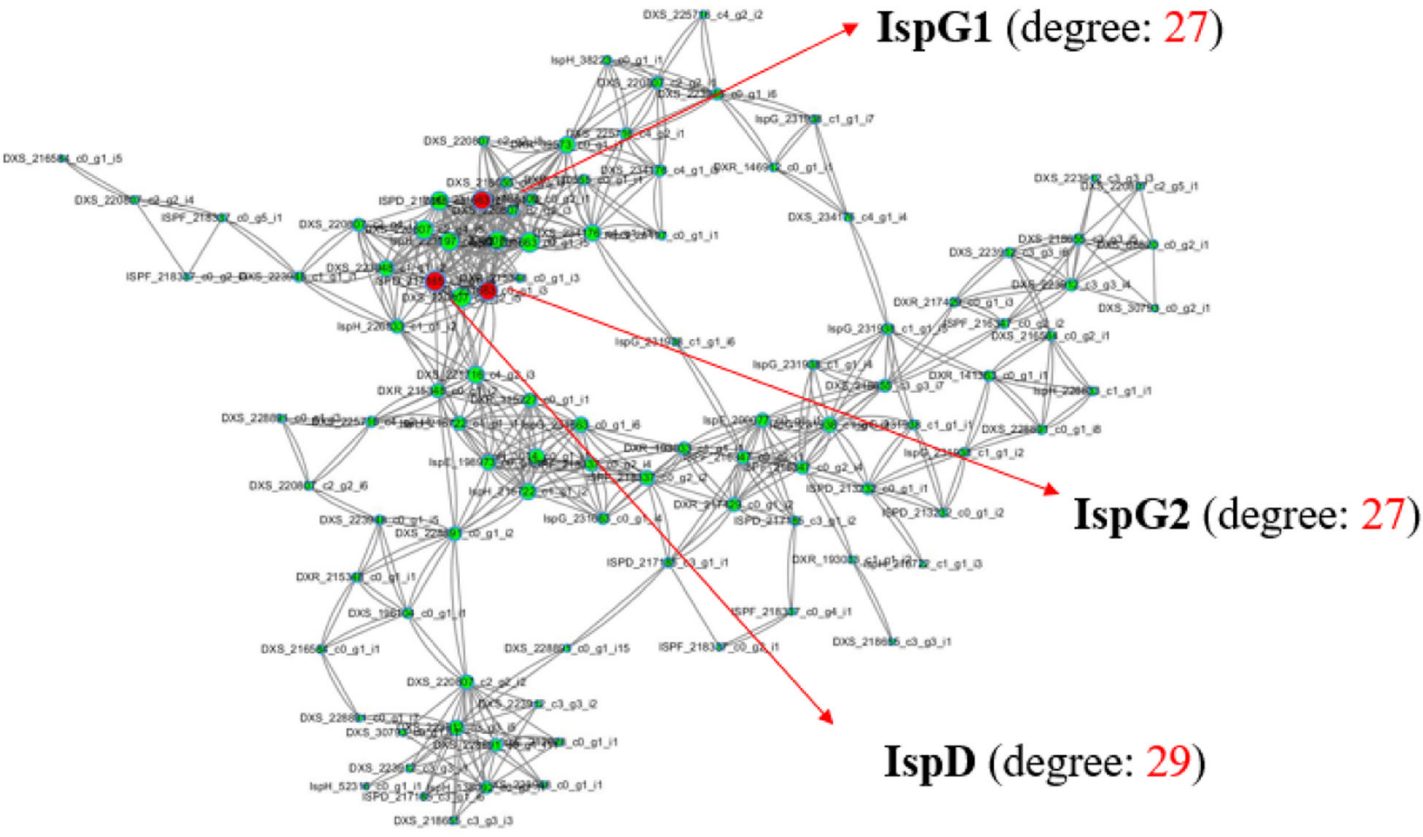
Co-expression network analysis diagram of the genes related to the MEP pathway in the root samples of 1–5 year-old *P. ginseng*. Totally 113 genes used in the co-expression network analysis using Cytoscape software version 3.6.0 (Spearman test cutoff value> = 0.9). IspD, IspE and IspG, the genes of the MEP pathway

To determine whether the IspD gene was the key element in the MEP pathway for the terpene precursor biosynthesis, we further carried out the co-expression network analysis on the genes (TPM > =1) of MEP pathway using RNA-seq data of the ginseng root samples of 1–5 years old (Fig. 5). The results showed that there were 113 nodes and 603 edges in the network. The average degree value was 11 and the max degree value was 29. The average of neighborhood connectivity was 7 and the max value was 14. Among them, IspD had the highest degree value, 29, implying that IspD was a key enzyme in the MEP pathway, consistent with the lowest transcription levels found in all the tissues of ginseng plants described above (Fig. 4b).

### Real-time PCR analysis

**Fig. 6:**
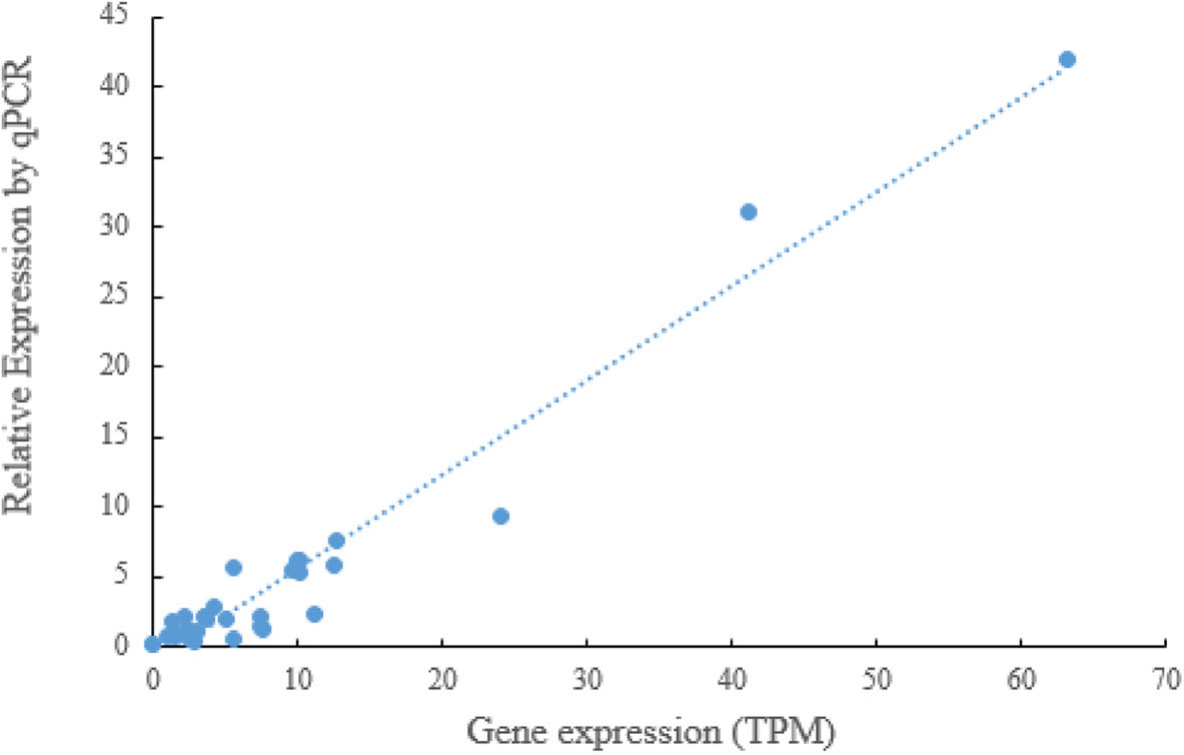
PCR verification on gene expression levels related to the MEP pathway of *P. ginseng* between RNA-seq analyses and qRT-PCR assays. Totally 30 ginseng tissue samples were randomly selected, including IspD gene, to carry out the qRT-PCR assays. The results were calculated by spearman test and the cutoff value is 0.8

Due to the high sensitivity of RNA sequencing technology, we finally examined the gene expression levels in different ginseng tissue samples by random selection of several genes of the MEP pathway, including IspD gene, to carry out quantitative RT-PCR (qPCR) validation experiments. The gene names and the corresponding primers were listed in Additional file 2: Table S2. The gene expression correlation between RNA-seq results and qPCR verification was shown in Fig. 6. The correlation coefficient (R) was as high as above 0.825, explaining the reliability of gene expression results in the RNA-seq data of ginseng samples in this study.

## Discussion

### De novo assembly of RNA-seq data without reference genome of *P. ginseng*

The powerful advancements of the next generation sequencing (NGS) technology, with reduced sequencing cost and improved computational skills, make RNA-seq methodology more widely used in the studies of disclosing gene expression and metabolic regulation among various species. Particularly for the medicinal plants without reference genome information due to their complex genome structures, RNA-seq studies across various tissues with different characteristics or phenotypes or with different treatments can provide valuable chances to sight in their gene structure and annotation, gene expression and function, gene phylogenetic evolution, metabolic pathways and regulation, as well as provide compensative information for their further genomic studies [27, 28].

When we constructed the gene sets based on our RNA-seq data of different tissue samples of *P. ginseng*, the ginseng’s genome sequencing results and a PacBio RNA-seq data had not been published until September 2017 [15, 16, 20]. Therefore we used the de novo method to assemble the transcribing genes based on our RNA-seq data. After assembly, we checked the transcribed genes in each samples and their expression distribution patterns were reasonable (Additional file 8: Figure S3). We finally compared our de novo-assembly gene sets with the above-mentioned three published datasets, named as IPGA [16], Renamed [20] (both sequenced base on Illumina Hiseq platforms) and Iso-seq (the RNA-seq assembled dataset sequenced using a PacBio platform, [15]) (Additional file 3: Table S3). Except for the Iso-seq data which had priority on the length of sequencing reads and assembly contigs, the N50 of our RNA-seq assembly contigs was 1811 bp, while those of the IPGA and the Renamed were 1446 bp and 1790 bp, respectively. The total unigene number of our RNA-seq assembly was 48,165, and those of the IPGA and the Renamed were 59,352 and 42,006, respectively. The average length of our RNA-seq assembly transcripts, the genomic CDS sequences of the IPGA and the Renamed was 1310 bp, 1120 bp and 1379 bp, respectively. The max length of our RNA-seq assembly isoforms, the genomic CDS sequences of the IPGA and the Renamed was 15,220 bp, 16,536 bp and 11,130 bp, respectively. Therefore, our ginseng’s RNA-seq assembly gene sets had similar results to the newly published genomic CDS gene sets based the NGS technology, indicating the high RNA-seq quality of our ginseng samples and reliable analysis results in this study.

We further compared our assembled datasets with the above-mentioned three published datasets based both on all the transcribed genes (Additional file 12: Figure S7) and those involved in the ginsenoside biosynthesis (Additional file 4: Table S4). The spearman correlation analyses between our RNA-seq assembled gene sets and three published datasets showed the average correlation indexes (R) were around 0.82–0.83 (Additional file 12: Figure S7), indicating the good quality of our de novo RNA-seq assembly. The comparison of annotation on the genes involved in the ginsenoside biosynthesis between our de novo assembled RNA-seq dataset and the three published datasets showed that the annotated gene numbers in our de novo assembled RNA-seq dataset were similar to those of the Renamed dataset, more than those of the Iso-seq dataset, and far more than those of the IPGA dataset (Additional file 4: Table S4). We also compared the annotated ginsenoside-biosynthesis gene sequences one by one between our de novo assembled RNA-seq dataset and three published datasets and found that for each gene, the annotated isoforms were multiple mapping each other between different datasets (data not shown due to huge data sheet). We also found some of the genes had quite different sequences base on different datasets. For example, the *IspD* gene of our de novo assembled RNA-seq dataset had similar DNA sequence to that of the IPGA dataset, but quite different from those of the Renamed dataset and the Iso-seq dataset, and even different between the Renamed dataset and the Iso-seq dataset (Additional file 13: Figure S8). The reason of such bias was not known and need further investigation. However, we finally proved the sequence of our *IspD* gene isoform by PCR (Additional file 2: Table S2 and Fig. 6). Therefore, we ultimately used our de novo assembled RNA-seq gene sets for analyses in this study.

We also compared ginsenoside biosynthesis-related gene annotation between our RNA-seq data and those of other close species of *Panax*, including *P. japonicus* [25], *P. quinquefolium* [29], as well as *Withania somnifera* [30]. The results showed that more gene isoforms were identified in both the MEP pathway and the MVA pathway of *P. ginseng*, but no significant difference in the triterpene skeleton synthesis and ginsenoside synthesis modules, except for IPI gene (Additional file 5: Table S5). Notably, the MVK gene of the MVA pathway and the IspD gene of the MEP pathway were identified with relatively fewer isoforms in each species, indicating that they might have greater impacts on the entire terpene-derived metabolic pathway. According to our RNA-seq data, along with the MVA pathway, the transcription levels of MVK gene and its following genes were obviously at relatively low levels in all the ginseng tissues, particularly in the leaves (Figs. 3b, d and 4a), while IspD gene of the MEP pathway significantly transcribed at very low levels in all the tissues (Figs. 3a, c and 4b) of *P. ginseng*. Therefore, it is worth to exam the MVK and IspD genes’ function in terpene precursor generation and the whole ginsenoside biosynthesis pathway in the following study. These two genes might be the new targets in the ginsenoside biosynthesis regulation in future’s synthetic biology study.

### Transcriptomic profiling of ginsenoside biosynthesis in *P. ginseng*

Due to the medicinal values of *P. ginseng*, its roots, leaves, stems and flowers have been extensively sustained RNA-seq studies [13, 14]. But our RNA-seq study launched comprehensive analyses not only on main mature ginseng tissues of 5 years old but also on ginseng roots from one to five years old, for ginseng plant’s medicinal part is usually its root tissues and the 5-year old ginseng plant is commonly regarded as mature enough to harvest for medicinal utility (Samukawa et a., 1995). Our RNA-seq study could better show the characteristics and changes of gene expressions relevant to both growth age and different tissues of ginseng plants. The results definitely revealed that the gene transcript abundances related to ginsenoside biosynthesis obviously began to increase since the age of 3 years old and up to the highest levels at 5 years old (Fig. 1a) when ginseng plants usually begin to be harvested in the cultivation conditions. Attentively, there was a gene-expression decrease detected in our RNA-seq data of the 4-year old ginseng root samples (Fig. 1a). It is interesting that Samukawa and coworkers also reported that ginsenoside contents in the cultivated ginseng roots in Nagano, Japan increased annually from 3 years old but decreased at the fourth year old and increased again at the fifth and the sixth years old (Samukawa et a.,1995). This coincidence proposed that ginsenoside biosynthesis might be easily influenced by the cultivation conditions or habitat environments, which needs more experiments and data to prove.

As to the underground ginseng tissues (lateral roots, root barks, root cores and rhizomes), our transcriptomic profiling revealed that all the ginsenoside biosynthesis-related genes transcribed at more abundances in the lateral roots/root barks and rhizomes than in the root cores (Fig. 1b). These results consisted with previous ginsenoside examination studies among various ginseng tissues [31]. For the aboveground ginseng tissues, the genes related to the MEP pathway and the third metabolic module for ginsenoside biosynthesis obviously transcribed at higher levels in the leaves than in the stems (Fig. 1b), indicating the plastid-origin of the MEP pathway, as well as GT and CYP450 super families transcribed mainly in the living cells but not in vascular or xylem cells of ginseng plants.

There are many researches reporting that different IPP-derived metabolites come from different IPP biosynthesis pathway, i.e. from the MVA pathway or the non-MVA pathway (i.e. MEP pathway) [19, 32]. For example, sterols, sesquiterpenes and ubiquinones are mainly synthesized through the MVA pathway, while hemi-, mono-, sesqui- and di- terpenes, carotenoids and phytol chains of chlorophyll are predominantly from the non-MVA pathway [32]. The differential expression patterns of the MVA pathway and the MEP pathway in the roots and leaves of *P. ginseng* indicated that the resources of terpene precursors for various ginsenosides and other IPP-derived metabolites biosynthesis might be quite different in ginseng leaves and roots (Fig. 1a, b). Such differentiation may result in different type ginsenosides accumulated in ginseng roots and leaves, and may also lead to tissue-specific P450 genes and GT genes occurred in ginseng roots or leaves accounting for tissue-specific ginsenoside generation. Recently Xu and colleges also found that the contents of ginsenosides Rg1, Re, Rf, Rg2, Rb1, Rc, Rb2, and Rd. were significantly higher in the periderm (root bark) than in the cortex and stele (root cortex and root core) using high performance liquid chromatography (HPLC) detection [20]. Therefore, further deep RNA-seq data mining with new sequencing strategy design is needed to provide more genomic information on ginsenoside biosynthetic-related gene function study.

### The MEP pathway plays essential roles in ginsenoside biosynthesis in ginseng tissues containing plastids or chloroplasts

Ginsenosides are triterpenoid saponins. It is widely accepted that they are synthesized from the terpene precursor, IPP and its isoform, DMAPP [13, 14, 17, 18]. Presently in plants, it has been well established that IPP is biosynthesized from both the cytosol MVA pathway and the plastic non-MVA (also named as MEP or DOXP) pathway [19]. The MVA pathway was first discovered as early as in the 1950s, but the MEP pathway was revealed more recently in 1990s [33]. It has been observed that some secondary compounds are synthesized exclusively through only one of the pathways, such as 2-methyl-3-buten-2-olsynthesis via the MEP pathway in needles of *Pinus ponderosa* [34], terpinolene and myrcene via MEP pathway in *Daucus carota* L. [35], (−)-R-pinene and (S)-linalool in raspberry fruits via the MVA pathway [36], and hydrocarbon, botryococcene, and tetramethylsqualene via the MEP pathway in the *Botryococcus braunii* race B [37]. However, in some cases both pathways play important roles, such as artemisinin in *Artemisia annua* L. [38], dolichols in *Coluria geoides* [39], gibberellins in *A. thaliana* [21], polyisoprenoids in *Eucommia ulmoides* [40], and polyisoprene (rubber) in *H. brasiliensis* [41]. Nevertheless, the question of whether both pathways or only one of them furnishes ginsenoside biosynthesis remains to be answered until Zhao and coworkers took advantage of the two pathway’s inhibitors using hairy root culture system of *P. ginseng* and provided biochemistry-experimental evidences to indicate that ginsenosides are synthesized through both the MVA and the MEP pathways and more preferably through the MVA pathway [19].

So far, the MVA pathway participated in the ginsenoside biosynthesis in *Panax* genus had been reported by many research based on both phytochemical experiments and transcriptome and genome sequencing studies [13, 14, 17, 18]. But there is no detail report about the MEP pathway in *P. ginseng* on transcriptomic levels except that Chen’s study found there were intermediates of the MEP pathway in ginseng genomic study [20]. Our study for the first time revealed that the genes related to the MEP pathway was mainly expressed in ginseng leaves, followed by in the lateral roots or root barks and lowest in the stems and root cores (Fig. 1a), as well as the genes in the MVA pathway and the MEP pathway had almost similar transcription abundances in general (Fig. 3a, b), indicating the MEP pathway also plays essential roles in ginsenoside biosynthesis in the tissues containing plastids or chloroplasts of *P. ginseng*. This finding is consistent with the plastic origin of the MEP pathway on evolution [9], and also consists with the results that most of the MEP pathway-related genes transcribed at higher in the leaves than in the roots in *A. thaliana* [8] and *H. brasiliensis* [41].

### IspD, the key gene of the MEP pathway

Our further gene expression and co-expression network analyses also indicated that IspD might be a rate-limiting enzyme in ginsenoside biosynthesis in *P. ginseng*. The first reason is that based on the whole gene expression profiles related to ginsenoside synthesis in our study, IspD had the lowest expression level among all the ginseng tissue samples (Fig. 4b), indicating that it might affect the subsequent IPP-derived metabolite production. The second reason is that the results of co-expression network analysis also referred to that IspD was a key gene in the MEP pathway (Fig. 4). The third reason is that there were fewer isoforms annotated on *IspD* gene (Table 2) and fewer transcript abundances (Fig. 3a, c and Fig. 4b) for the *IspD* gene compared with other genes in the MEP pathway in our ginseng RNA-seq study. Therefore, we speculated that IspD might quite possibly be a key enzyme in the ginsenoside biosynthesis in *P. ginseng*. Gao and colleges had reported that IspD was also the key enzyme in the MEP pathway in *Mycobacterium tuberculosis*, which causes tuberculosis in humans, and was selected as a chemo- therapeutic target to inhibit the mycobacterium growth [42]. Our results suggested that IspD might be a potential target for metabolic manipulation of ginsenoside biosynthesis in *P. ginseng* using the synthetic biological technology.

## Conclusions

All the genes encoding enzymes in the MEP pathway (DXS, DXR, IspD, IspE, IspF, IspG and IspH) were identified in *P. ginseng* based on our deep RNA-seq analyses. We further illustrated the comprehensive gene expression profiles related to ginsenoside biosynthesis, particularly including the MEP pathway, among1–5 year-old roots and different tissues of 5 year-old plants of *P. ginseng.* We for the first time revealed that gene transcription abundances of the MEP pathway were similar to those of the MVA pathway in ginseng roots but higher in ginseng leaves. We predicted that IspD was the key enzyme in the MEP pathway. These results provided solid transcriptional bioinformation for further study on metabolic regulation on various ginsenoside production using synthetic biology techniques.

## Materials and methods

### Plant materials

The 1–5 years old ginseng plants were collected in field at the ginseng farm in Jilin Province in northeast of China in late August. After rinsing with distilled water immediately to clean the soil, the ginsengs’ main root barks (periderm tissue in the main roots), main root cores (vascular and xylem tissue in the main roots), lateral roots (diameter between 1 and 3 mm), root hairs (diameter less than 1 mm), rhizomes, stems and leaves were separately cut into small pieces quickly, packaged in small pieces of aluminum foils and immediately frozen in the liquid nitrogen for storage until further processing. The root tissues used in this study are the lateral roots or main root barks of *P. ginseng*. The photographs of *P. ginseng* plants collected in the field and the dissected ginseng tissues used in this study were shown in Additional 6: Figure S1.

### RNA extraction

Total RNA was extracted from each tissue sample using TRIzol reagent (Life Technologies, Ambion, New Zealand) and digested with DNase I (Waryong, Beijing, China) according to the manufacturer’s protocol. Finally, oligo (dT) magnetic beads (Kapa Biosystems, USA) were used to isolate mRNA from the total RNA. By mixing with fragmentation buffer, the mRNA was then broken into short fragments.

### Sequencing library construction and sequencing

The cDNA was synthesized using the mRNA fragments as templates. The short fragments were purified and resolved with the Elution buffer for end repair and single nucleotide A addition, and then connected with adapters. Target fragments were selected as templates for PCR amplification to construct the cDNA sequencing libraries (KAPA Hyper Prep, Kapa Biosystems, USA). Each cDNA library was sequenced by an Illumina HiSeq 2000/HiSeq 3000 system using paired end protocols according to the manufacturer’s instruction at the Beijing Institute of Genomics, Chinese Academy of Sciences.

### De novo assembly sequences

After sequencing, the raw reads datasets were evaluated by a FastQC software (https://www.bioinformatics.babraham.ac.uk/projects/fastqc/). The adapter sequences, lower quality reads (that were shorter than 30 bp or quality score lower than 20), ribosomal RNA sequences, mitochondrial and chloroplast genomic sequences and bacterial sequences were all filtered to obtain the high-quality mRNA sequencing reads (Additional file 1: Table S1). The high-quality reads from all samples were merged to do de novo assembly using the Trinity software v2.2.0 [43] to get an assembly gene set. The longest transcript of the same genes was selected as the unigene.

All of the raw reads generated in this study have been deposited in NCBI and can be accessed in the Sequence Read Achieve (SRA) Sequence Database under the bioproject accession number SRP151182.

### Annotation and expression calculation of the genes related to saponin biosynthesis

The conserved domain region of each enzyme related to saponin biosynthesis in *Arabidopsis* was identified with the Interproscan software [44] from *Arabidopsis* genome database (https://www.arabidopsis.org/) and the longest sequence was screened and retrieved to group together as a reference gene set. The assembled ginseng RNA-seq data were BLASTx against this reference gene set to annotate the genes that participate in the ginsenoside biosynthesis of *P. ginseng*. Then the annotated genes were verified by BLASTx against non-redundant protein (Nr) databases (ftp://ftp.ncbi.nih.gov/blast/db/FASTA/nr.gz) of NCBI with a cut-off E-value of 10^− 5^.

All the annotated and verified genes associated with saponin biosynthesis in each ginseng RNA-seq sample were retrieved. The gene expression level of each enzyme was calculated by summarize all of its gene expression values together and expressed as TPM (Transcripts Per Million) using RSEM package [45]. To calculate the gene expression level of each metabolic pathway, we summarized all the gene expression values of all the enzymes of the whole pathway. To calculate the gene expression level of each ginsenoside biosynthesis module, all the gene expression values belonging to the same module were summarized. The gene expression patterns of each enzyme, each metabolic pathway or each module among 1–5-year old root samples or different 5-year-old tissue samples were clustered using R package heatmap2based on their relative expression levels.

### Correlation assay of the biological repeat samples

The gene expression correlation between two biological replicates of root samples of 1–5 years old *P. ginseng* was analyzed by using pearson test (*p* value <=0.05 was regarded as to have significant correlation).

### Co-expression network analysis of MEP pathway

In order to find the key enzymes of the MEP pathway for terpene precursor production, we constructed a co-expression network analysis map using Cytoscape software [46] based on the correlation of the gene expression levels of the MEP pathway in 1–5-year old ginseng root samples (spearman test cutoff value> = 0.9). The obtained core gene was further to predict its functional protein association network using STRING online tools (https://string-db.org/).

### Real-time PCR assay

Total RNA isolation and cDNA preparation from different tissue samples of *P. ginseng* were the same as those above in “RNA extraction” and “Sequencing library construction”. Quantitative PCR reaction were performed on the Real-Time PCR Detection System (ABI 7500, Applied Biosystems, USA) using SYBRR Premix Ex Taq™ II (TOYOBO, Japan). For each reaction, 1 μl (0.5 μM) of the forward and the reverse primers and 2 ng of cDNA template were added. All the primers used in this study are listed in Additional file 2: Table S2. The relative gene expression level was calculated according to the 2^-ΔΔCt^ method [47]. For each sample, the mRNA abundances of the target genes were normalized to those of beta actin gene. These experiments were repeated with three biological replications. Finally, the gene expression correlations between RNA-seq analyses and qPCR experimental results were calculated by spearman test.

## Additional files

Additional file 1: **Table S1.** Statistics of quality control on RNA-seq data of 1–5 years old root samples and other tissue samples of 5 years old of *P. ginseng. (PDF 68 kb)*

Additional file 2: **Table S2.** Primer sequences of the genes of the MEP pathway of *P. ginseng* for qPCR verification (PDF 57 kb)

Additional file 3: **Table S3.** Comparison of ginseng CDS gene sets between published genomic dataset and our assembled RNA-seq dataset of *P. ginseng (PDF 66 kb) (PDF 66 kb)*

Additional file 4: **Table S4.** Comparison analyses on the gene numbers annotated in ginsenoside biosynthesis-related pathways between our assembled RNA-seqdataset and other published datasets. (PDF 50 kb)

Additional file 5: **Table S5.** Statistics of the isoform numbers of the genes related to ginsenoside biosynthesis among different Panax species and *W. somnifera* based on RNA-seq data. (PDF 90 kb)

Additional file 6: **Figure S1.** Photographs of *P. ginseng* plants collected in the field and the dissected ginseng tissues used in this study (including main root barks, main root cores, lateral roots, root hairs, rhizomes, stems and leaves). A, the photograph of *P. ginseng* root. B, dissections of a ginseng root to show the tissues of main roots, small roots (diameter between 2 mm and 3.5 mm), lateral roots (diameter between 1 mm and 2 mm), root hairs (diameter less than 1 mm) and rhizomes. C, dissections of a ginseng root to show the tissues of the main root bark and main root cores. D. 1 year to 5 years old ginseng plants collected in the field. The scale bar was indicated in each pattern. (PDF 130 kb)

Additional file 7: **Figure S2.** Saturation curve of transcriptome sequencing data of each samples of *P. ginseng* using BWA software. R11 and R12, two biological duplication samples of 1 year-old root samples. R21 and R22, two biological duplication samples of 2 year-old root samples. R3a and R35, two biological duplication samples of 3 year-old root samples. R41 and R42, two biological duplication samples of 4 year-old root samples. R52 and R5b, two biological duplication samples of 5 year-old root samples. Rc, root cores. Rt, lateral roots. Bs, rhizomes. St, stems. Lf, leaves. (PDF 125 kb)

Additional file 8: **Figure S3.** Histograms of gene transcription abundance distribution of RNA-seq samples of *P. ginseng* using RSEM software. R11 and R12, two biological duplication samples of 1 year-old root samples. R21 and R22, two biological duplication samples of 2 year-old root samples. R3a and R35, two biological duplication samples of 3 year-old root samples. R41 and R42, two biological duplication samples of 4 year-old root samples. R52 and R5b, two biological duplication samples of 5 year-old root samples. Rc, root cores. Rt, lateral roots. Bs, rhizomes. St, stems. Lf, leaves. (PDF 66 kb)

Additional file 9: **Figure S4.** Correlation analyses of the gene expression of the MEP pathway between the two biological duplication samples of 1–5 years old *P. ginseng*. The analyses were calculated using R Studio software and person test with cutoff value of 0.8. R1-R5, two biological duplication samples of 1–5 year-old root samples. (PDF 60 kb)

Additional file 10: **Figure S5.** The expression profile and clustering analyses on differential expressed genes (TPM > 0) of the MEP pathway in the biological duplication root samples of 1–5 year-old *P. ginseng*. The analyses were calculated using RSEM software, and the cutoff value is 1.0. R11 and R12, two biological duplication samples of 1 year-old root samples. R11 and R12, two biological duplication samples of 1 year-old root samples. R21 and R22, two biological duplication samples of 2 year-old root samples. R3a and R35, two biological duplication samples of 3 year-old root samples. R41 and R42, two biological duplication samples of 4 year-old root samples. R52 and R5b, two biological duplication samples of 5 year-old root samples. (PDF 51 kb)

Additional file 11: **Figure S6.** The transcript abundances of each gene’s isoforms were variant and changed in different tissues (A) and in 1–5 years old roots (B) during ginseng growing up. (PDF 245 kb)

Additional file 12: **Figure S7.** Spearman correlation analysis on our assembled datasets and three published datasets, the IPGA, the Renamed and the Iso-seq, based on all the transcribed genes. (*p* value <=0.05) (PDF 37 kb)

Additional file 13: **Figure S8.** The alignment among IspD isoforms obtained from the IPGA dataset (isoform No. 1), the Renamed dataset (isoform No. 2) and our de novo TRINITY assembly dataset (isoform No. 10–17), based on the Illumina HiSeq platforms, as well as from the Iso-seq dataset (isoform No. 3–9) based on the PacBio sequencing platform. The result showed that two isoforms of our de novo TRINITY assembly (isoform No. 10–11) had high identity with that of IPGA (isoform No. 1). But other isoforms of our de novo TRINITY assembly had little similarity with that of the Renamed dataset and Iso-seq dataset. (PDF 597 kb)

## Abbreviations

AACT: Acetyl-CoA C-acetyltransferase
CDS: Coding sequence
DMAPP: Dimethylallyl diphosphate
DS: Dammarenediol synthase
DXR: 1-deoxy-D-xylulose-5-phosphate reductoisomerase
DXS: 1-deoxy-D-xylulose-5-phosphate synthase
FPP: Farnesyl diphosphate
FPS: Farnesyl diphosphate synthase
GT: Glycosyltransferase
HMGR: 3-hydroxy-3-methylglutaryl-CoA reductase
HMGS: 3-hydroxy-3-methylglutaryl-CoA synthase
HPLC: High-performance liquid chromatography
IDI: Isopentenyldiphosphate delta-isomerase
IPP: Isopentenyl diphosphate
IspD: 2-C-methyl-D-erythritol 4-phosphate cytidylyltransferase
IspE: 4-(Cytidine 5-diphospho)-2-C-methyl-D-erythritol kinase
IspF: 2-C-methyl-D-erythritol 2,4-cyclodiphosphate synthase
IspG: 4-Hydroxy-3-methylbut-2-enyl-diphosphate synthase
IspH: 4-Hydroxy-3-methylbut-2-enyl diphosphate reductase
MEP: 2-C-methyl-D-erythritol 4-phosphate
MVA: Mevalonic acid
MVD: Mevalonate diphosphate decarboxylase
MVK: Mevalonate kinase
MVP: Mevalonate phosphate
MVPP: Diphosphomevalonate
PMK: Phosphomevalonate kinase
PPD: Protopanaxadiol
PPDS: Protopanaxadiol synthase
PPT: Protopanaxatriol
PPTS: Protopanaxatriol synthase
SE: Squalene epoxidase
SQS: Squalene synthase
SS: Squalene synthase
UDP: Uridinediphosphate
UGT: UDP-glycosyltransferase
*β*-AS: *β*-amyrin synthase
