# Supplementary material for: Transcriptomic profiling reveals MEP pathway contributing to ginsenoside biosynthesis in Panax ginseng
Source: BMC Genomics. 2019 May 17;20:383. doi: 10.1186/s12864-019-5718-x (PMC6524269; doi:10.1186/s12864-019-5718-x)
Supplement: Supplementary file 1 — Table S1. Statistics of quality control on RNA-seq data of 1–5 years old root samples and other tissue samples of 5 years old of P. ginseng. (PDF 68 kb) [file 12864_2019_5718_MOESM1_ESM.pdf]

Additional Table S1 Statistics of quality control on RNA-seq data of 1-5 years old root samples and other tissue samples of 5 years old plants of *P. ginseng*

Sample	Raw data	Raw reads	Filtered reads	Percentage	Nuclear clear reads	Percentage
R1-1	9.4G	40644422	40482612	99.60%	39648794	97.55%
R1-2	9.2G	40400776	38952660	96.42%	38558192	95.44%
R2-1	5.2G	20643126	20548220	99.54%	18698010	90.58%
R2-2	7.0G	28472356	27238486	95.67%	25411870	89.25%
R3-5	26G	100979214	100783574	99.81%	96467786	95.53%
R3-a	24G	95546958	94119296	98.51%	93020350	97.36%
R4-1	8.0G	34802958	34717464	99.75%	34505262	99.14%
R4-2	8.0G	34553094	34489114	99.81%	34260124	99.15%
R5-2	10.4G	45579794	45467100	99.75%	45034216	98.80%
R5-b	18.6G	76921546	76750900	99.78%	75689192	98.40%
Root	10.4G	45018762	40102174	89.08%	38610788	85.77%
Rhizome	7.6G	33343654	32106432	96.29%	31555328	94.64%
Leaf	9.0G	38872596	34801638	89.53%	33551056	86.31%
Stem	9.4G	40743924	39583584	97.15%	34812458	85.44%
Root core	6.6G	27190970	26928878	99.04%	25881132	95.18%

Note: R11 and R12, two biological duplication samples of 1 year-old root samples. R21 and R22, two biological duplication samples of 2 year-old root samples. R3a and R35, two biological duplication samples of 3 year-old root samples. R41 and R42, two biological duplication samples of 4 year-old root samples. R52 and R5b, two biological duplication samples of 5 year-old root samples. Root, lateral root samples. Root core, vascular and xylem tissue in ginseng roots.
