# Supplementary material for: Transcriptomic profiling reveals MEP pathway contributing to ginsenoside biosynthesis in Panax ginseng
Source: BMC Genomics. 2019 May 17;20:383. doi: 10.1186/s12864-019-5718-x (PMC6524269; doi:10.1186/s12864-019-5718-x)
Supplement: Supplementary file 2 — Table S2. Primer sequences of the genes of the MEP pathway of P. ginseng for qPCR verification (PDF 57 kb) [file 12864_2019_5718_MOESM2_ESM.pdf]

Additional Table S2 Primer sequences of the genes of the MEP pathway of *P. ginseng* for qPCR verification

Primer	sequence (5' to 3')
DXR-F	ATAACACCCACCATCCACTCC
DXR-R	TTCTGCCCATTTGACATACC
DXS-F	TTCTGCTGGCTTAGGAATGG
DXS-R	TGGCTGTAGGTAGGGAGACTTG
IspD-F	AGAAGGATCACAAACAACAACG
IspD-R	TTTAGCAGGAGGAAAAGGCA
IspE-F	AAGGTCAAATGAAATAGGTGGG
IspE-R	TACAGGGAAAAGACGGGC
IspF-F	GTGGGGGCTTAGTTTTGGTC
IspF-R	TGATGCGATTTTGGGTGC
IspG-F	CTCGTTTTACCACCGTCTTCC
IspG-R	TGTCTCATGCCCATCTTGCG
IspH-F	GTTTTCTTTCTCAACCAACTCG
IspH-R	AAGCAACACCTCGCACCTA
beta-actin-F	GAAAGCCGAACAGAGAAAGG
beta-actin-R	TGAGGAATATGGGGCAAGTATA
