# Supplementary material for: Transcriptomic profiling reveals MEP pathway contributing to ginsenoside biosynthesis in Panax ginseng
Source: BMC Genomics. 2019 May 17;20:383. doi: 10.1186/s12864-019-5718-x (PMC6524269; doi:10.1186/s12864-019-5718-x)
Supplement: Supplementary file 3 — Table S3. Comparison of ginseng CDS gene sets between published genomic dataset and our assembled RNA-seq dataset of P. ginseng (PDF 66 kb) (PDF 66 kb) [file 12864_2019_5718_MOESM3_ESM.pdf]

Additional Table S3 Comparison of ginseng CDS gene sets between published genomic and transcriptomic datasets and our assembled RNA-seq dataset of *P. ginseng*

	CDS_version of our RNA-seq assembly (accession No. SRP151182)	CDS_version of the IPGA (http://ginsengdb.snu.ac.kr/data.php)[a]	CDS_version of the Renamed (http://ginseng.vicp.io:23488/)[b]	CDS_version of Iso-seq[c]
N50 (bp)	1811	1446	1790	3745
N90 (bp)	660	555	756	1902
Total numbers	48165	59352	42006	135317
Total length (bp)	63117625	66492402	57939557	430008043
Average length (bp)	1310	1120	1379	3177
Max length (bp)	15220	16536	11130	21843
Min length (bp)	201	153	178	300
Median length (bp)	1048	885	1193	3009

Note: References used in this table: a, the genomic data of Kim et al. Plant Biotechnology Journal, 2018, 1–14. b, the genomic data of Xu et al. GigaScience, 2017, 6, 1 –15. C, the PacBio transcriptomic-seq data of Jo et al. Genes 2017, 8, 228.
