# Supplementary material for: Transcriptomic profiling reveals MEP pathway contributing to ginsenoside biosynthesis in Panax ginseng
Source: BMC Genomics. 2019 May 17;20:383. doi: 10.1186/s12864-019-5718-x (PMC6524269; doi:10.1186/s12864-019-5718-x)
Supplement: Supplementary file 4 — Table S4. Comparison analyses on the gene numbers annotated in ginsenoside biosynthesis-related pathways between our assembled RNA-seqdataset and other published datasets. (PDF 50 kb) [file 12864_2019_5718_MOESM4_ESM.pdf]

**Additional Table S4 Comparison analyses on the gene annotations involved in MEP/MVA/ginsenoside biosynthesis-related pathways between our assembled RNA-seq dataset and two published reference genomes and one Pacbio transcriptome of *P. ginseng*.**

| Pathway | Enzyme | Our-assembled | Iso-seq | IPGA | Renamed |
|---|---|---|---|---|---|
| MEP | DXR | 14 | 4 | - | 9 |
| | DXS | 59 | 20 | 1 | 43 |
| | IspD | 8 | 1 | 1 | 7 |
| | IspE | 6 | 7 | - | 23 |
| | IspF | 10 | 4 | - | 2 |
| | IspG | 14 | 6 | 2 | 6 |
| | IspH | 11 | 4 | 2 | 4 |
| MVA | AACT | 20 | 12 | - | 29 |
| | HMGR | 34 | 15 | 1 | 34 |
| | HMGS | 23 | 5 | 1 | 7 |
| | MVD | 2 | 5 | - | 8 |
| | MVK | 13 | 3 | - | 9 |
| | PMK | 30 | 125 | 14 | 37 |
| Skeleton | FPS | 15 | 3 | 1 | 16 |
| | GGR | 33 | 12 | 1 | 27 |
| | IDI | 6 | 2 | - | 5 |
| | SS | 13 | 3 | 2 | 7 |
| Saponin | AS | 18 | 5 | - | 34 |
| | DS | 7 | 3 | 1 | 4 |
| | SE | 22 | 6 | - | 18 |
| | GT | 260 | 27 | 9 | 65 |
| | P450 | 221 | 66 | 8 | 151 |

Note: The annotation threshhold was set as the identity>=90% and gene transcription abundance (TPM) >=1. "-" means not to be annotated.

The reference transcriptomic data of Iso-seq (Jo et al. Genes 2017, 8, 228), genomic data of IPGA (Xu et al. GigaScience, 2017, 6, 1-15) and Renamed (Kim et al. Plant Biotechnology Journal, 2018, 1-14 ) were cited in the table.
