# Supplementary material for: Transcriptomic profiling reveals MEP pathway contributing to ginsenoside biosynthesis in Panax ginseng
Source: BMC Genomics. 2019 May 17;20:383. doi: 10.1186/s12864-019-5718-x (PMC6524269; doi:10.1186/s12864-019-5718-x)
Supplement: Supplementary file 5 — Table S5. Statistics of the isoform numbers of the genes related to ginsenoside biosynthesis among different Panax species and W. somnifera based on RNA-seq data. (PDF 90 kb) [file 12864_2019_5718_MOESM5_ESM.pdf]

Additional Table S5 Statistics of the isoform numbers of the genes related to ginsenoside biosynthesis among different *Panax* species and *W. somnifera* based on RNA-seq data

pathway	gene	*P. ginseng*	*P. quinquefolium*	*P. japonicus*	*W. somnifera*
**MVA**	AACT	102	20	18	15
	HMGS	46	17	10	26
	HMGR	47	20	19	23
	MVK	4	8	4	4
	PMK	31	12	20	11
	MVD	8	6	8	8
**MEP**	DXS	99	51	34	47
	DXR	16	7	8	13
	IspD	10	4	6	6
	IspE	18	21	13	17
	IspF	26	17	8	16
	IspG	29	16	13	8
	IspH	48	19	4	12
**Skeleton**	IPI	12	2	2	27
	GGR	36	47	49	48
	FPS	12	8	10	44
	SS	8	14	18	18
**Saponin**	SE	38	37	14	34
	beta-AS	14	116	54	46
	DS	4	58	28	22
	GT	200	204	195	356
	CYP450	243	430	410	689

Note: W. somnifera, Withania Somnifera. SRA accession numbers of the public RNA-seq datasets used in above table: SRR2164754 (one year-old leaf), SRR1586196 (seed), SRR1182934 (leaf) of P. quinquefolium. SRR3203822 (flower), SRR3203826 (lateral root), SRR3203825 (young rhizome), SRR3203824 (old rhizome), SRR3203823 (leaf) of 7 year-old Panax japonicus C. A. Mey. SRR1197746 (leaf) and SRR1197573 (root) of W. somnifera. The annotation threshhold was TPM >=1.
