# Supplementary figures and images for: Transcriptomic profiling reveals MEP pathway contributing to ginsenoside biosynthesis in Panax ginseng
Source: BMC Genomics. 2019 May 17;20:383. doi: 10.1186/s12864-019-5718-x (PMC6524269; doi:10.1186/s12864-019-5718-x)

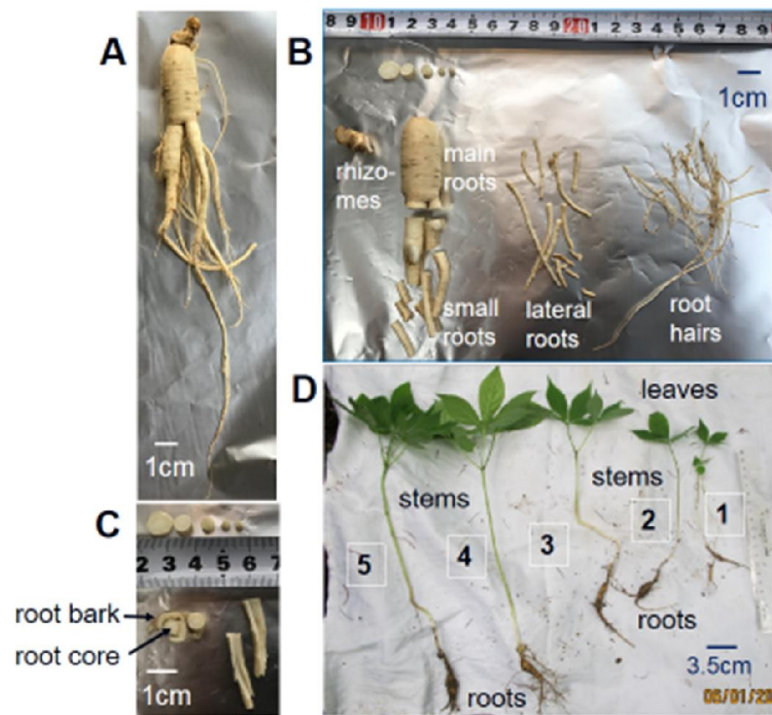

Additional Figure S1

Supplement: Supplementary file 6 — Figure S1. Photographs of P. ginseng plants collected in the field and the dissected ginseng tissues used in this study (including main root barks, main root cores, lateral roots, root hairs, rhizomes, stems and leaves). A, the photograph of P. ginseng root. B, dissections of a ginseng root to show the tissues of main roots, small roots (diameter between 2 mm and 3.5 mm), lateral roots (diameter between 1 mm and 2 mm), root hairs (diameter less than 1 mm) and rhizomes. C, dissections of a ginseng root to show the tissues of the main root bark and main root cores. D. 1 year to 5 years old ginseng plants collected in the field. The scale bar was indicated in each pattern. (PDF 130 kb) [file 12864_2019_5718_MOESM6_ESM.pdf]

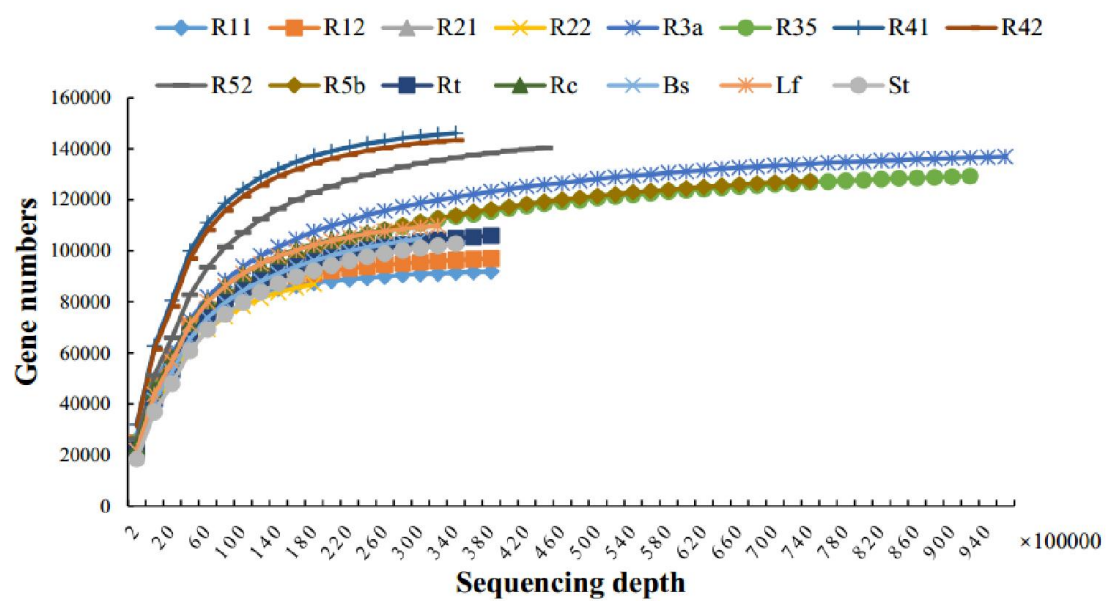

Additional Figure S2

Supplement: Supplementary file 7 — Figure S2. Saturation curve of transcriptome sequencing data of each samples of P. ginseng using BWA software. R11 and R12, two biological duplication samples of 1 year-old root samples. R21 and R22, two biological duplication samples of 2 year-old root samples. R3a and R35, two biological duplication samples of 3 year-old root samples. R41 and R42, two biological duplication samples of 4 year-old root samples. R52 and R5b, two biological duplication samples of 5 year-old root samples. Rc, root cores. Rt, lateral roots. Bs, rhizomes. St, stems. Lf, leaves. (PDF 125 kb) [file 12864_2019_5718_MOESM7_ESM.pdf]

A

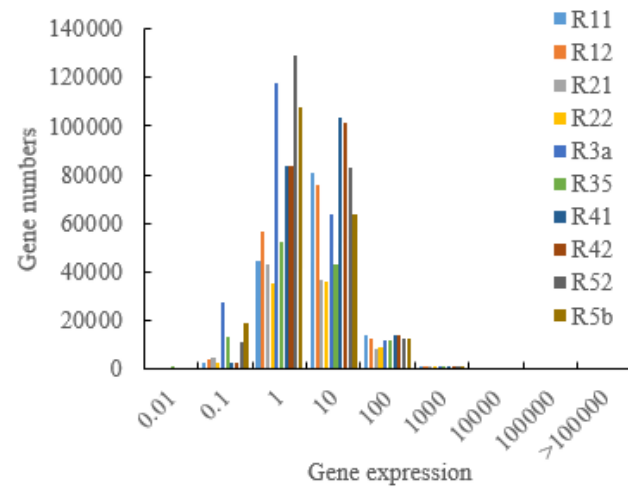

B

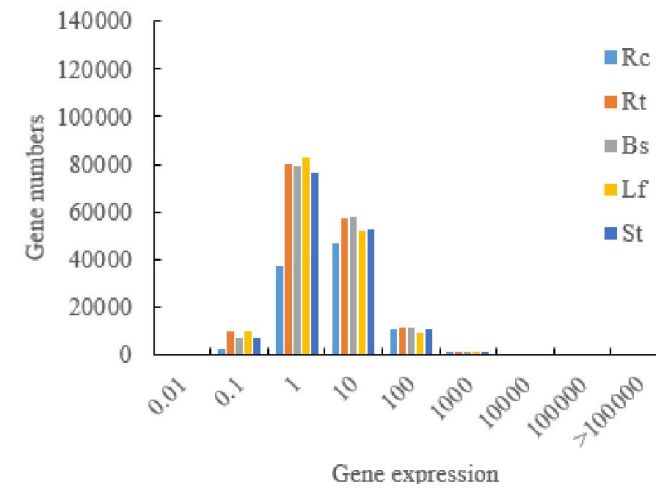

Additional Figure S3

Supplement: Supplementary file 8 — Figure S3. Histograms of gene transcription abundance distribution of RNA-seq samples of P. ginseng using RSEM software. R11 and R12, two biological duplication samples of 1 year-old root samples. R21 and R22, two biological duplication samples of 2 year-old root samples. R3a and R35, two biological duplication samples of 3 year-old root samples. R41 and R42, two biological duplication samples of 4 year-old root samples. R52 and R5b, two biological duplication samples of 5 year-old root samples. Rc, root cores. Rt, lateral roots. Bs, rhizomes. St, stems. Lf, leaves. (PDF 66 kb) [file 12864_2019_5718_MOESM8_ESM.pdf]

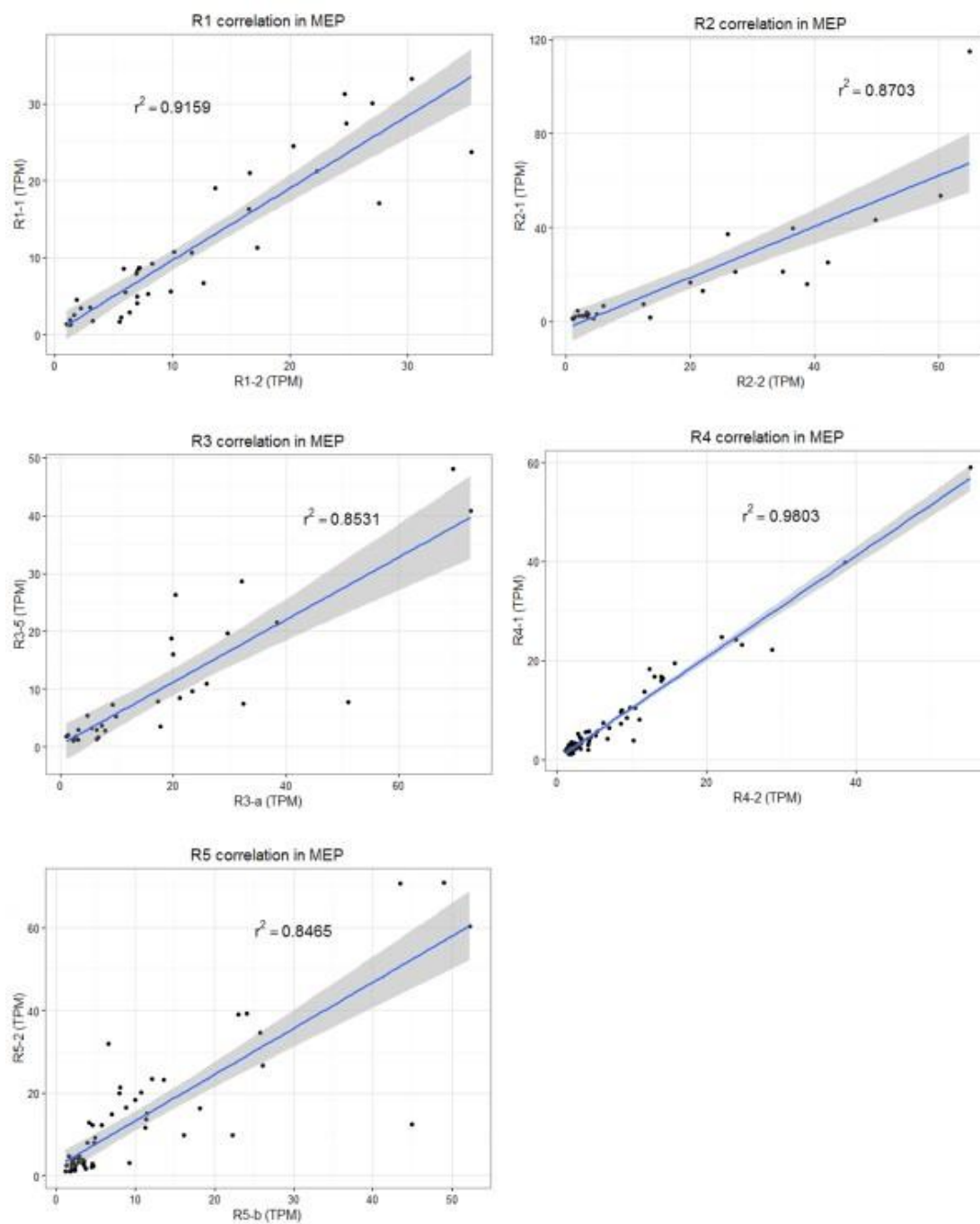

Additional Figure S4

Supplement: Supplementary file 9 — Figure S4. Correlation analyses of the gene expression of the MEP pathway between the two biological duplication samples of 1–5 years old P. ginseng. The analyses were calculated using R Studio software and person test with cutoff value of 0.8. R1-R5, two biological duplication samples of 1–5 year-old root samples. (PDF 60 kb) [file 12864_2019_5718_MOESM9_ESM.pdf]

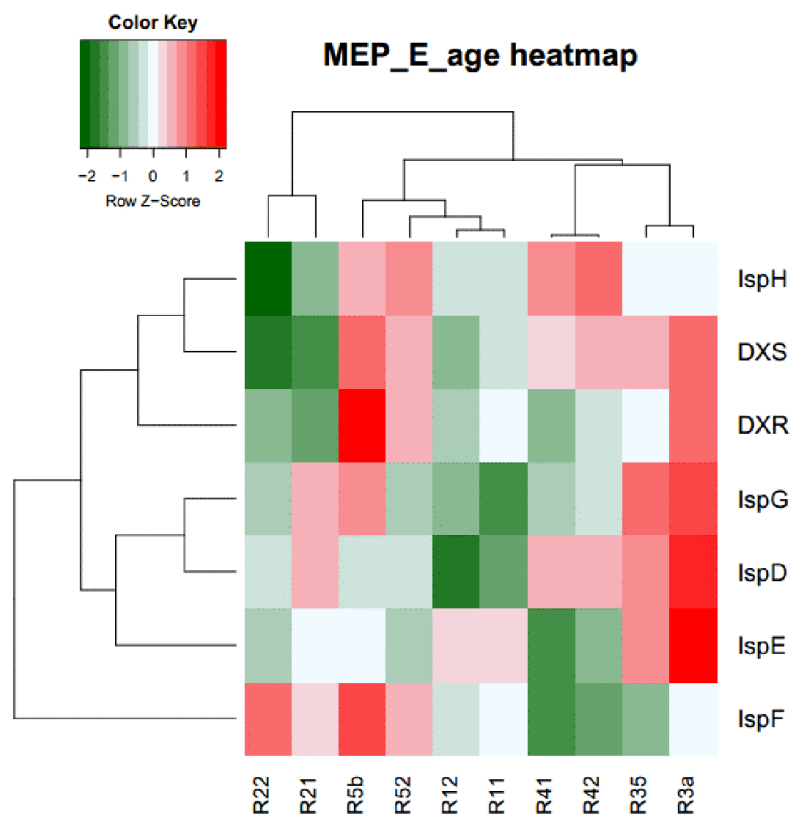

Additional Figure S5

Supplement: Supplementary file 10 — Figure S5. The expression profile and clustering analyses on differential expressed genes (TPM > 0) of the MEP pathway in the biological duplication root samples of 1–5 year-old P. ginseng. The analyses were calculated using RSEM software, and the cutoff value is 1.0. R11 and R12, two biological duplication samples of 1 year-old root samples. R11 and R12, two biological duplication samples of 1 year-old root samples. R21 and R22, two biological duplication samples of 2 year-old root samples. R3a and R35, two biological duplication samples of 3 year-old root samples. R41 and R42, two biological duplication samples of 4 year-old root samples. R52 and R5b, two biological duplication samples of 5 year-old root samples. (PDF 51 kb) [file 12864_2019_5718_MOESM10_ESM.pdf]

## A MEP pathway in 1-5 years old toots

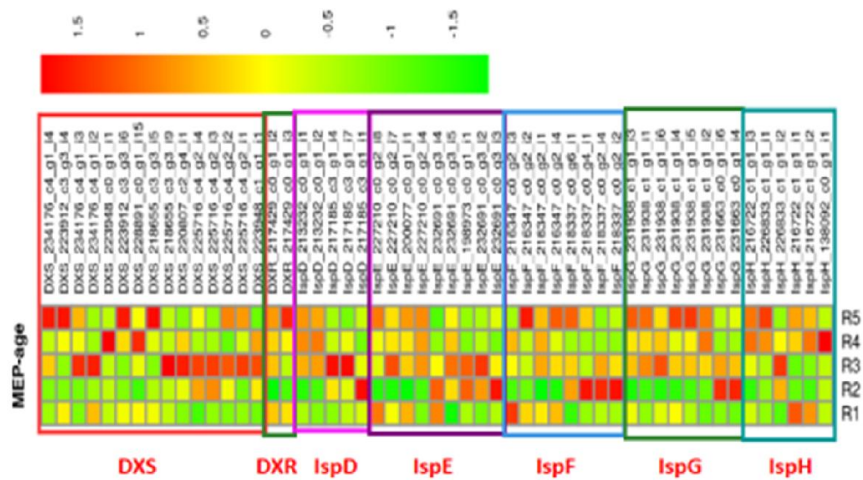

## B MEP pathway in different tissues

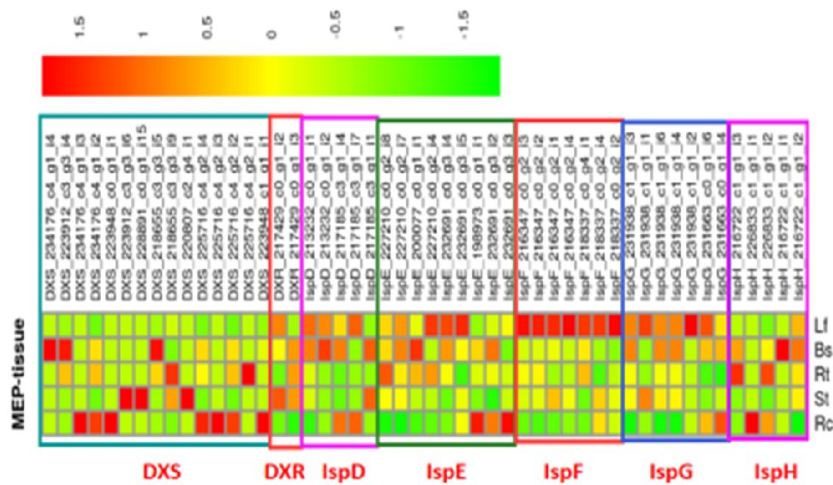

Additional Figure S6

Supplement: Supplementary file 11 — Figure S6. The transcript abundances of each gene’s isoforms were variant and changed in different tissues (A) and in 1–5 years old roots (B) during ginseng growing up. (PDF 245 kb) [file 12864_2019_5718_MOESM11_ESM.pdf]

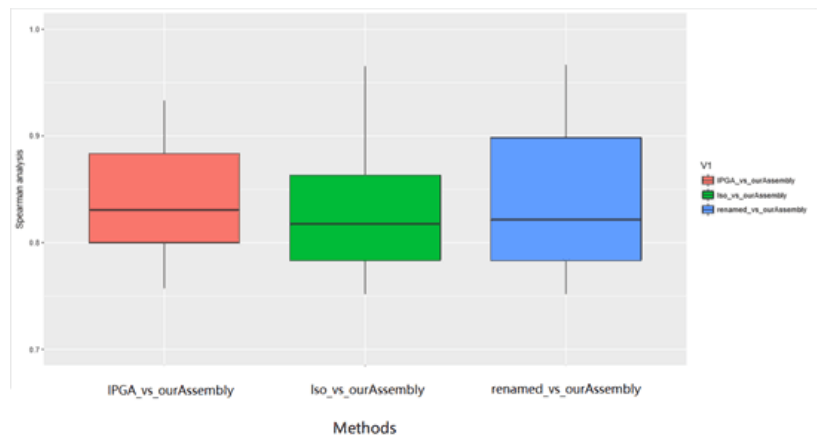

Additional Figure S7

Supplement: Supplementary file 12 — Figure S7. Spearman correlation analysis on our assembled datasets and three published datasets, the IPGA, the Renamed and the Iso-seq, based on all the transcribed genes. (p value <=0.05) (PDF 37 kb) [file 12864_2019_5718_MOESM12_ESM.pdf]
