# Supplementary material for: Transcriptomic profiling reveals MEP pathway contributing to ginsenoside biosynthesis in Panax ginseng
Source: BMC Genomics. 2019 May 17;20:383. doi: 10.1186/s12864-019-5718-x (PMC6524269; doi:10.1186/s12864-019-5718-x)
Supplement: Supplementary file 13 — Figure S8. The alignment among IspD isoforms obtained from the IPGA dataset (isoform No. 1), the Renamed dataset (isoform No. 2) and our de novo TRINITY assembly dataset (isoform No. 10–17), based on the Illumina HiSeq platforms, as well as from the Iso-seq dataset (isoform No. 3–9) based on the PacBio sequencing platform. The result showed that two isoforms of our de novo TRINITY assembly (isoform No. 10–11) had high identity with that of IPGA (isoform No. 1). But other isoforms of our de novo TRINITY assembly had little similarity with that of the Renamed dataset and Iso-seq dataset. (PDF 597 kb) [file 12864_2019_5718_MOESM13_ESM.pdf]

Species/Abbrv	Group Name	
1. Pg_S0544.32_IPGA		--
2. PG36909-mRNA-1_Renamed		--
3. KG_ISO_081828_ISO		--
4. KG_ISO_089664_ISO		--
5. KG_ISO_100437_ISO		--
6. KG_ISO_101107_ISO		--
7. KG_ISO_110390_ISO		--
8. KG_ISO_115245_ISO		ATTTTTCCTTTTAACTTAAAAACGTTGTTTCTCTATTTATTCAACCCTCTGTCATCGGACGCTACCTCAGATTTTCACACAA
9. KG_ISO_123515_ISO		--AGATTTTCACACAA
10. TRINITY_DN213232_c0_g1_i1		--
11. TRINITY_DN213232_c0_g1_i2		--
12. TRINITY_DN217185_c3_g1_i1		--
13. TRINITY_DN217185_c3_g1_i4		--
14. TRINITY_DN217185_c3_g1_i7		--
15. TRINITY_DN217185_c3_g1_i2		--
16. TRINITY_DN217185_c3_g1_i3		--
17. TRINITY_DN217185_c3_g1_i6		--

Species/Abbrv	Group Name	
1. Pg_S0544.32_IPGA		--
2. PG36909-mRNA-1_Renamed		--
3. KG_ISO_081828_ISO		--
4. KG_ISO_089664_ISO		---------------------------AACTGAACTGAACACATTTTGAGATTTGGAAGATGAAGGCGAGAATGAAGATGAG
5. KG_ISO_100437_ISO		---GTCCA
6. KG_ISO_101107_ISO		--
7. KG_ISO_110390_ISO		---GAGATTTGGAAGATGAAGGCG-GAATGAAGATGAG
8. KG_ISO_115245_ISO		GCCTTAGCGAGTAGGTTGAATAATTGTTGTTCGTGTTTATCTGATCTTAGTTTCTGTATATCTGTTTG--AATGAATAAAAA
9. KG_ISO_123515_ISO		GCCTTAGCGAGTAGGTTGAATAATTGTTGTTCGTGTTTATCTGATCTTAGTTTCTGTATATCTGTTTG--AATGAATAAAA-
10. TRINITY_DN213232_c0_g1_i1		--
11. TRINITY_DN213232_c0_g1_i2		--
12. TRINITY_DN217185_c3_g1_i1		--
13. TRINITY_DN217185_c3_g1_i4		--
14. TRINITY_DN217185_c3_g1_i7		--
15. TRINITY_DN217185_c3_g1_i2		--
16. TRINITY_DN217185_c3_g1_i3		--
17. TRINITY_DN217185_c3_g1_i6		--

Species/Abbrv	Group Name	
1. Pg_S0544.32_IPGA		--
2. PG36909-mRNA-1_Renamed		--
3. KG_ISO_081828_ISO		--
4. KG_ISO_089664_ISO		GTTTCCACCTT-CGCAATCGGCGGCG-AAATCAG-----GCCCTTAAATGTGCTTCCCCAGGAATCATCCACAGTGCTAAAA
5. KG_ISO_100437_ISO		GTGTGGATGATGTCAATCTAATTTACTAGTAACTATAGACATCCAATTATTCTTTGTTTTTTTAATCTGCATATCCACAATA
6. KG_ISO_101107_ISO		--AGAGAC
7. KG_ISO_110390_ISO		GTTTCCACCTT-CGCAATCGGCGGCGGAAATCAG-----GCCCTTAAATGTGCTTCCCCAGGAATCATCCGCA-TGCTAAAA
8. KG_ISO_115245_ISO		GTTAGAACTCAGTACCATTGGTAAAAGACATAAGCGGAGAACTGTGTAAGCATTTACCGTGTAAGCATTTACCGTAGTATCT
9. KG_ISO_123515_ISO		GTTAGAACTCAGTACCATTGGTAAAAGACATAAGCGGAGAACTGTGTAAGCATTTACCGTGTAAGCATTTACCGTAGTATCT
10. TRINITY_DN213232_c0_g1_i1		--
11. TRINITY_DN213232_c0_g1_i2		--
12. TRINITY_DN217185_c3_g1_i1		--
13. TRINITY_DN217185_c3_g1_i4		--
14. TRINITY_DN217185_c3_g1_i7		--
15. TRINITY_DN217185_c3_g1_i2		--
16. TRINITY_DN217185_c3_g1_i3		--
17. TRINITY_DN217185_c3_g1_i6		--

Species/Abbrv	Group Name	
1. Pg_S0544.32_IPGA		--
2. PG36909-mRNA-1_Renamed		--
3. KG_ISO_081828_ISO		TTGATCATTGAATTTTTAATTAATTGATTTTTAAACTCAAAATTGACATAAAATCTTTTAATTTTAATCAATACAATGAATA
4. KG_ISO_089664_ISO		GAACAGTTATTGTTGTTATAATGTTAAGTATCAAAAACAGATTATCTTCCATGTTTCATCTGGTTGACACCTGAAACTTGAG
5. KG_ISO_100437_ISO		CTGCTCATTAGTTTGTATCTATACATAGTTTCCACACTCAGATCTTTTTTATCGTGCATTAGTTTCCCATACAAGACGTAAA
6. KG_ISO_101107_ISO		TTATATATATAGAGAGAGAGAGACATGGCATCAAAAT--ATTCAAGCAGTGGTGGCGGGAGTGGGAGGATTAGAGTGGGGAG
7. KG_ISO_110390_ISO		--
8. KG_ISO_115245_ISO		TTTGTTATGTGCTAACCCTGATCAGTGTCATCAAAATTCAGAAGCTCCTAGTTGGGAATTATTCTGAGATTGCCTTCTCTTG
9. KG_ISO_123515_ISO		TTTGTTATGTGCTAACCCTGATCAGTGTCATCAAAATTCAGAAGCTC-TAGTTGGGAATTATTCTGAGATTGCCTTCTCTTG
10. TRINITY_DN213232_c0_g1_i1		--
11. TRINITY_DN213232_c0_g1_i2		--
12. TRINITY_DN217185_c3_g1_i1		--
13. TRINITY_DN217185_c3_g1_i4		--
14. TRINITY_DN217185_c3_g1_i7		--
15. TRINITY_DN217185_c3_g1_i2		--
16. TRINITY_DN217185_c3_g1_i3		--
17. TRINITY_DN217185_c3_g1_i6		--

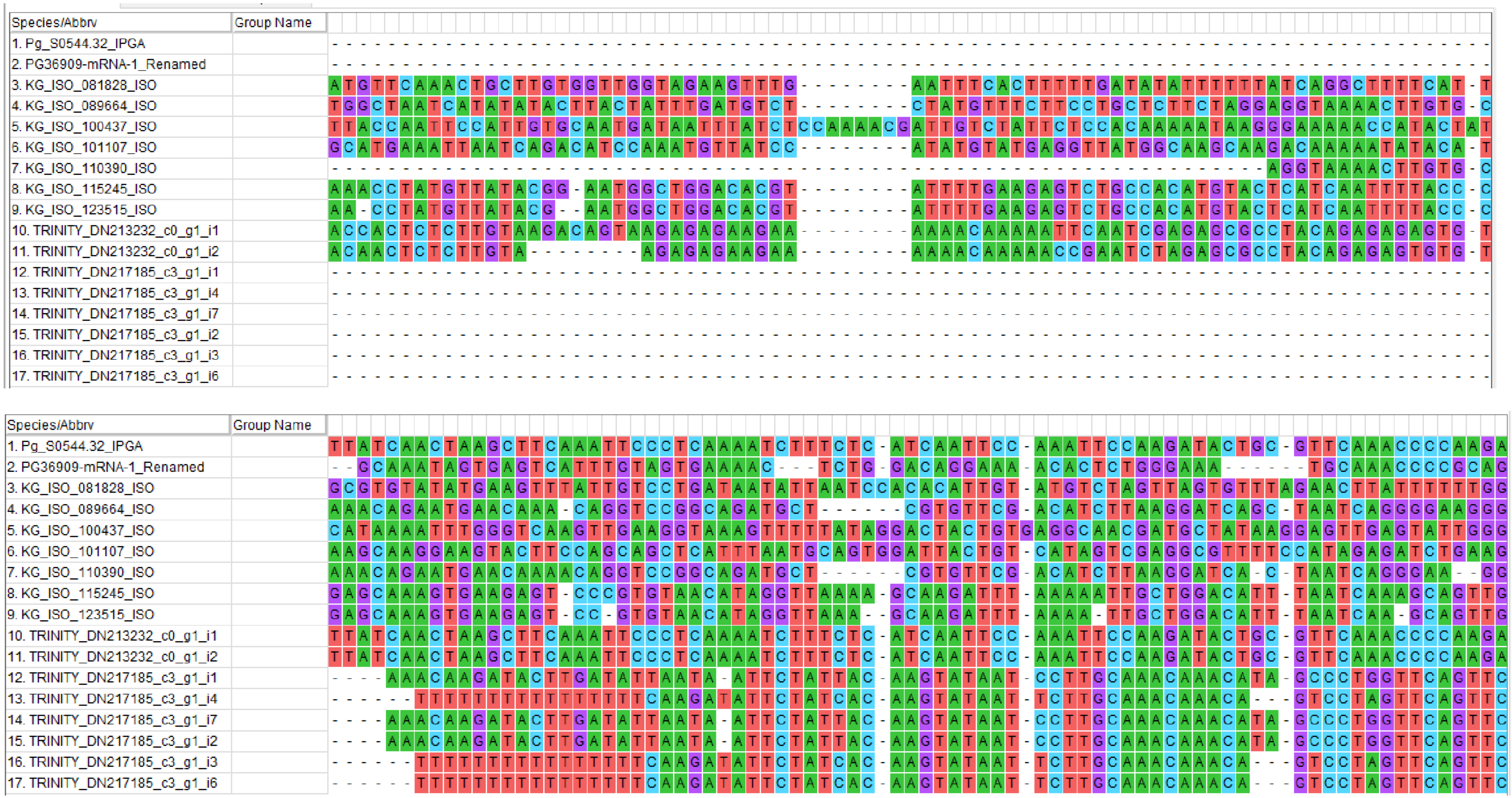
Species/Abbrv
Group Name
1. Pg_S0544.32_IPGA
2. PG36909-mRNA-1_Renamed
3. KG_ISO_081828_ISO
4. KG_ISO_089664_ISO
5. KG_ISO_100437_ISO
6. KG_ISO_101107_ISO
7. KG_ISO_110390_ISO
8. KG_ISO_115245_ISO
9. KG_ISO_123515_ISO
10. TRINITY_DN213232_c0_g1_i1
11. TRINITY_DN213232_c0_g1_i2
12. TRINITY_DN217185_c3_g1_i1
13. TRINITY_DN217185_c3_g1_i4
14. TRINITY_DN217185_c3_g1_i7
15. TRINITY_DN217185_c3_g1_i2
16. TRINITY_DN217185_c3_g1_i3
17. TRINITY_DN217185_c3_g1_i6

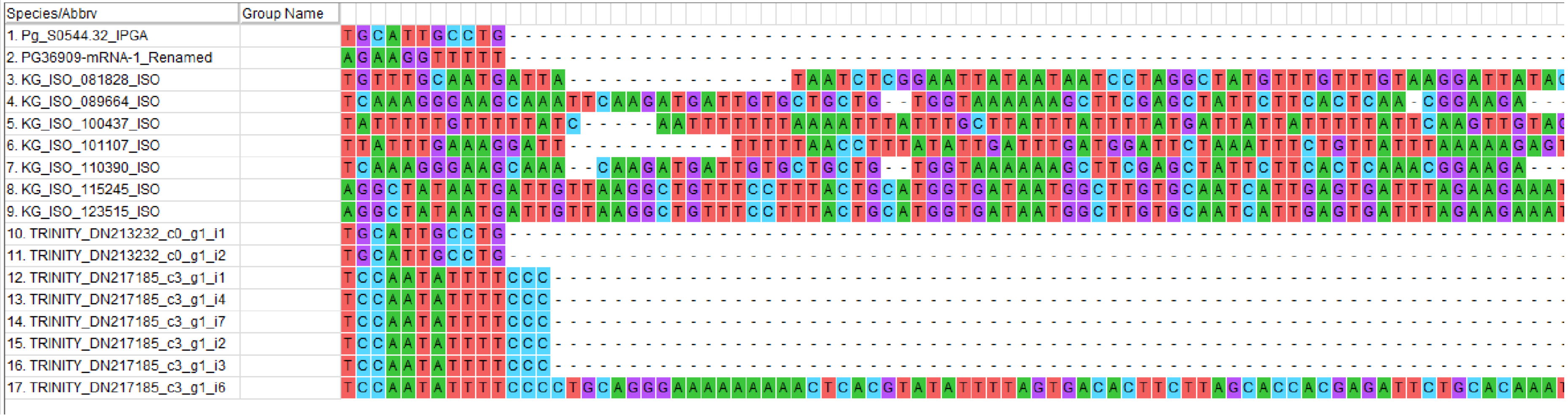
Species/Abbrv
Group Name
1. Pg_S0544.32_IPGA
2. PG36909-mRNA-1_Renamed
3. KG_ISO_081828_ISO
4. KG_ISO_089664_ISO
5. KG_ISO_100437_ISO
6. KG_ISO_101107_ISO
7. KG_ISO_110390_ISO
8. KG_ISO_115245_ISO
9. KG_ISO_123515_ISO
10. TRINITY_DN213232_c0_g1_i1
11. TRINITY_DN213232_c0_g1_i2
12. TRINITY_DN217185_c3_g1_i1
13. TRINITY_DN217185_c3_g1_i4
14. TRINITY_DN217185_c3_g1_i7
15. TRINITY_DN217185_c3_g1_i2
16. TRINITY_DN217185_c3_g1_i3
17. TRINITY_DN217185_c3_g1_i6

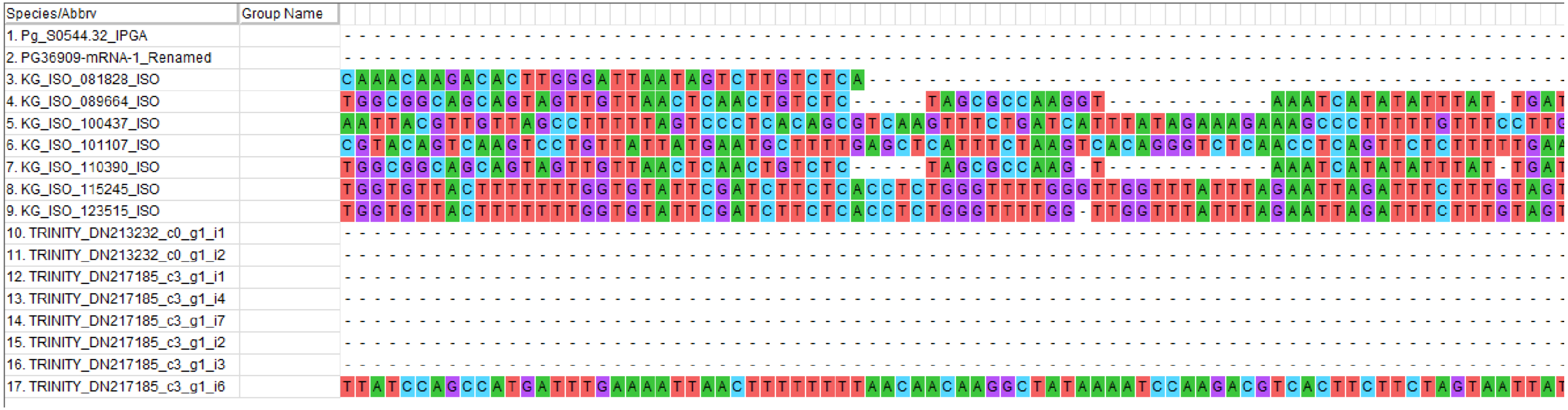
Species/Abbrv
Group Name
1. Pg_S0544.32_IPGA
2. PG36909-mRNA-1_Renamed
3. KG_ISO_081828_ISO
4. KG_ISO_089664_ISO
5. KG_ISO_100437_ISO
6. KG_ISO_101107_ISO
7. KG_ISO_110390_ISO
8. KG_ISO_115245_ISO
9. KG_ISO_123515_ISO
10. TRINITY_DN213232_c0_g1_i1
11. TRINITY_DN213232_c0_g1_i2
12. TRINITY_DN217185_c3_g1_i1
13. TRINITY_DN217185_c3_g1_i4
14. TRINITY_DN217185_c3_g1_i7
15. TRINITY_DN217185_c3_g1_i2
16. TRINITY_DN217185_c3_g1_i3
17. TRINITY_DN217185_c3_g1_i6

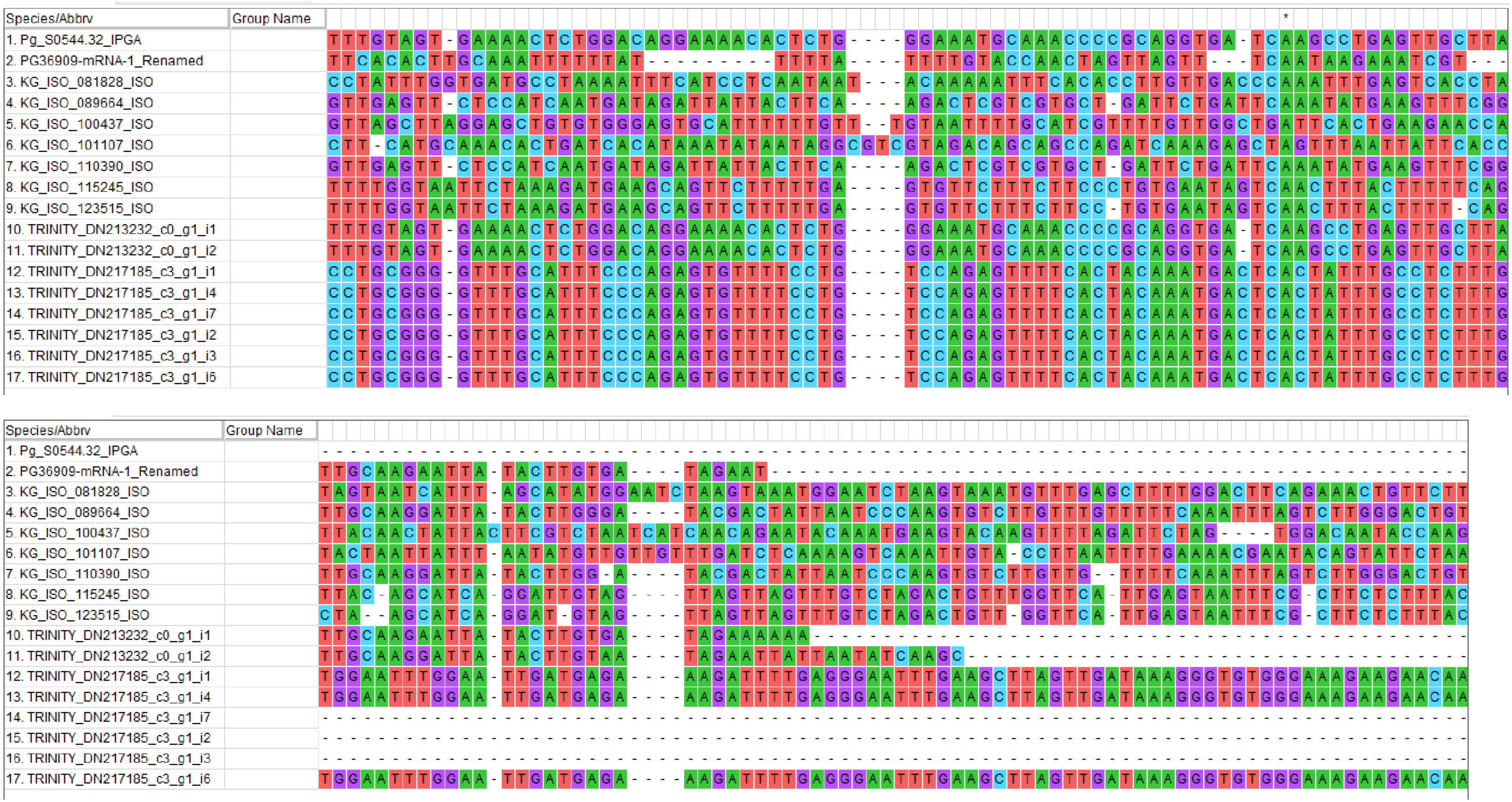
Species/Abbrv
Group Name
1. Pg_S0544.32_IPGA
2. PG36909-mRNA-1_Renamed
3. KG_ISO_081828_ISO
4. KG_ISO_089664_ISO
5. KG_ISO_100437_ISO
6. KG_ISO_101107_ISO
7. KG_ISO_110390_ISO
8. KG_ISO_115245_ISO
9. KG_ISO_123515_ISO
10. TRINITY_DN213232_c0_g1_i1
11. TRINITY_DN213232_c0_g1_i2
12. TRINITY_DN217185_c3_g1_i1
13. TRINITY_DN217185_c3_g1_i4
14. TRINITY_DN217185_c3_g1_i7
15. TRINITY_DN217185_c3_g1_i2
16. TRINITY_DN217185_c3_g1_i3
17. TRINITY_DN217185_c3_g1_i6

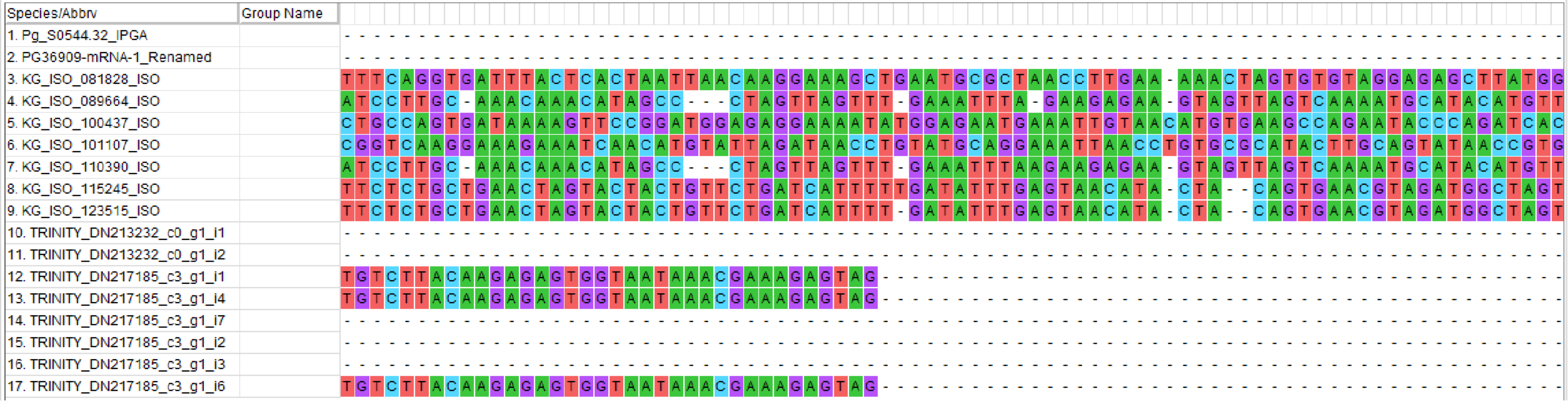
Species/Abbrv
Group Name
1. Pg_S0544.32_IPGA
2. PG36909-mRNA-1_Renamed
3. KG_ISO_081828_ISO
4. KG_ISO_089664_ISO
5. KG_ISO_100437_ISO
6. KG_ISO_101107_ISO
7. KG_ISO_110390_ISO
8. KG_ISO_115245_ISO
9. KG_ISO_123515_ISO
10. TRINITY_DN213232_c0_g1_i1
11. TRINITY_DN213232_c0_g1_i2
12. TRINITY_DN217185_c3_g1_i1
13. TRINITY_DN217185_c3_g1_i4
14. TRINITY_DN217185_c3_g1_i7
15. TRINITY_DN217185_c3_g1_i2
16. TRINITY_DN217185_c3_g1_i3
17. TRINITY_DN217185_c3_g1_i6

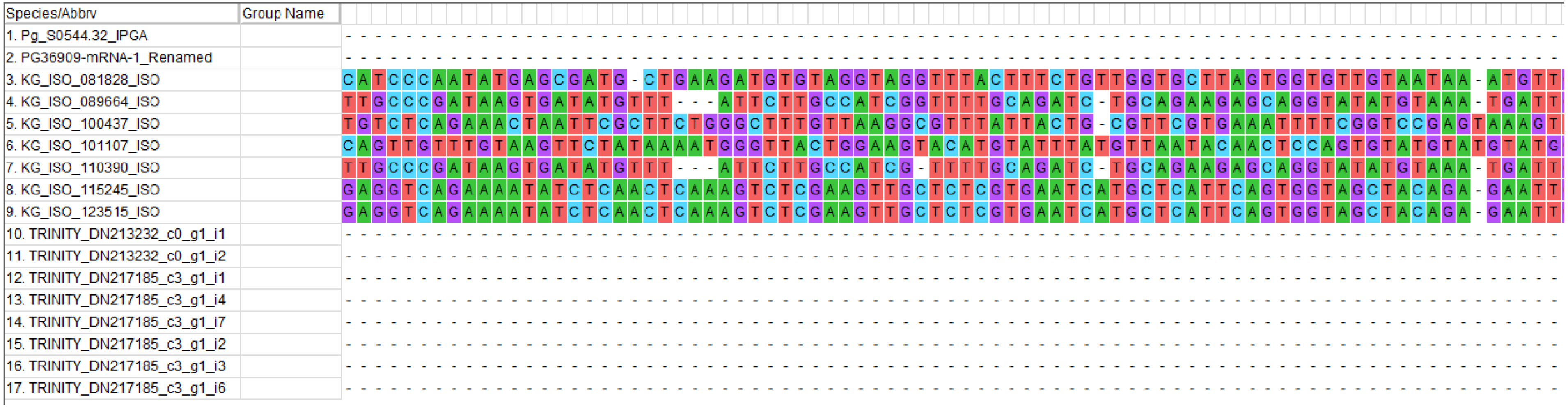
Species/Abbrv
Group Name
1. Pg_S0544.32_IPGA
2. PG36909-mRNA-1_Renamed
3. KG_ISO_081828_ISO
4. KG_ISO_089664_ISO
5. KG_ISO_100437_ISO
6. KG_ISO_101107_ISO
7. KG_ISO_110390_ISO
8. KG_ISO_115245_ISO
9. KG_ISO_123515_ISO
10. TRINITY_DN213232_c0_g1_i1
11. TRINITY_DN213232_c0_g1_i2
12. TRINITY_DN217185_c3_g1_i1
13. TRINITY_DN217185_c3_g1_i4
14. TRINITY_DN217185_c3_g1_i7
15. TRINITY_DN217185_c3_g1_i2
16. TRINITY_DN217185_c3_g1_i3
17. TRINITY_DN217185_c3_g1_i6

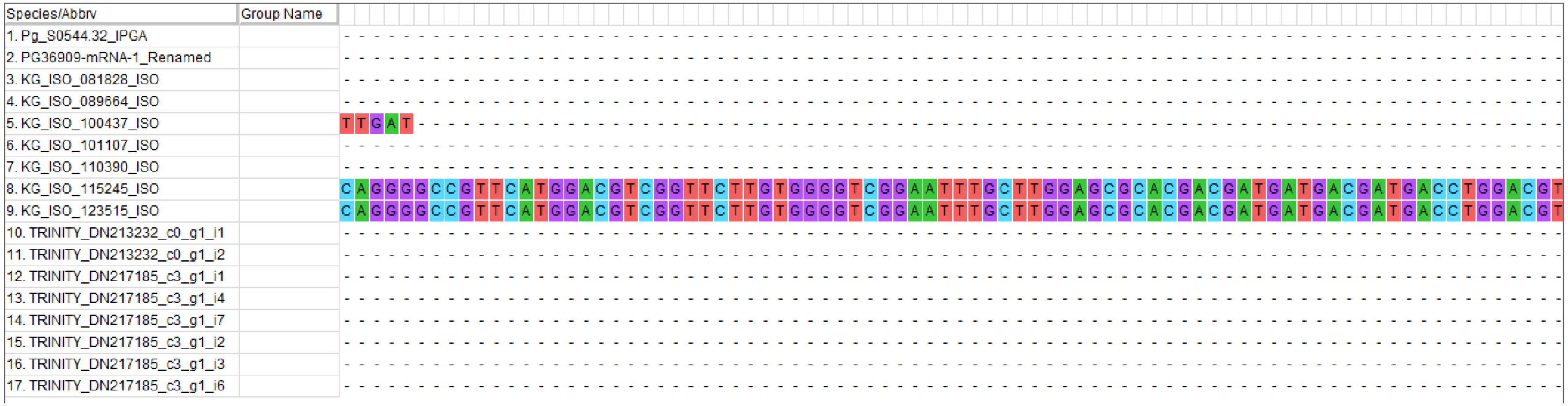

Additional Figure S8
